# Sensing and responding to allergic response cytokines through a genetically encoded circuit

**DOI:** 10.1038/s41467-017-01211-1

**Published:** 2017-10-24

**Authors:** Hélène Chassin, Barbara Geering, Lina Schukur, David Ausländer, Brian Lang, Martin Fussenegger

**Affiliations:** 1 Department of Biosystems Science and Engineering, ETH Zürich, Mattenstrasse 26, CH-4058 Basel, Switzerland; 2 Faculty of Science, University of Basel, Mattenstrasse 26, CH-4058 Basel, Switzerland; 3 Present Address: Pharma Research & Early Development, Roche, CH-4070 Basel, Switzerland

## Abstract

While constantly rising, the prevalence of allergies is globally one of the highest among chronic diseases. Current treatments of allergic diseases include the application of anti-histamines, immunotherapy, steroids, and anti-immunoglobulin E (IgE) antibodies. Here we report mammalian cells engineered with a synthetic signaling cascade able to monitor extracellular pathophysiological levels of interleukin 4 and interleukin 13, two main cytokines orchestrating allergic inflammation. Upon activation of transgenic cells by these cytokines, designed ankyrin repeat protein (DARPin) E2_79, a non-immunogenic protein binding human IgE, is secreted in a precisely controlled and reversible manner. Using human whole blood cell culturing, we demonstrate that the mammalian dual T helper 2 cytokine sensor produces sufficient levels of DARPin E2_79 to dampen histamine release in allergic subjects exposed to allergens. Hence, therapeutic gene networks monitoring disease-associated cytokines coupled with in situ production, secretion and systemic delivery of immunomodulatory biologics may foster advances in the treatment of allergies.

## Abstract (web summary)

The standard treatment for an allergic response is anti-histamines, steroids and anti-IgE antibodies. Here the authors present a genetic circuit that senses IL-4 and IL-13 and responses with DARPin production to bind IgE.

## Introduction

Allergies are reactions of the immune system toward foreign substances that do not cause immunogenicity under healthy conditions. The development of allergies is often inherited^1^, but it can be influenced by other factors, such as exposure to allergens during early life, the environment, and lifestyle^2–4^. In the early phase of an allergic reaction, allergens are recognized by professional antigen-presenting cells (APCs)^5^. These cells are able to take up and display fragments of allergens on major histocompatibility complex (MHC) class II molecules, which they carry on their cell surface^6^. Naive T cells can recognize the fragments via T-cell receptors, which leads to their differentiation into effector T helper 2 (T_H_2) cells^7^. These specialized effector cells produce and release several cytokines, including interleukin 4 (IL-4). IL-4 serves as an autocrine growth and differentiation factor^8^ and is responsible for the class switching of B cells to immunoglobulin E (IgE) synthesis^9^. Mast cells and basophil granulocytes bear high-affinity receptors for IgE (FcεRI) and bind free IgE in blood or tissue^10^. Pre-formed IgE–FcεRI complexes permit a rapid response by mast cells and basophil granulocytes: the allergens directly bind to the IgE on the surface, thereby promoting the aggregation of the IgE–FcεRI receptor complexes and triggering the intracellular inflammatory cascade^11, 12^. Subsequently, an immediate release of soluble mediators promotes the allergic inflammation. These mediators consist of pre-formed and newly synthesized compounds including histamine, cytokines, chemokines and leukotrienes^13^. Among the cytokines released after FcεRI aggregation, interleukin 13 (IL-13) plays a major role in the development of atopic asthmatic disease^14, 15^. IL-13, along with epidermal, neural, vascular and fibroblast growth factors, drives the production of mucus and is responsible for the remodeling of airway walls^16^. Elevated amounts of IL-13 can cause excessive production of mucus thereby narrowing the airways and increasing the typical asthmatic symptoms^17, 18^. Once the early phase of the allergic reaction has developed into a chronic allergic inflammation, the production of IL-4 and IL-13 occurs in a positive feedback loop, which results in an increase in IgE levels^19^.

Various attempts have been made to develop potent treatments against allergies. Many options include either the neutralization of histamine or the prevention of its release^20^. Histamine is produced by mast cells and basophil granulocytes and is responsible for the main symptoms of allergic diseases^21, 22^. Allergen-specific immunotherapy, another form of treatment, consists of the desensitization of the immune system by administration of appropriate concentrations of allergen extracts^23^. Of all these approaches, the direct targeting of the IgE molecule appears to be the most promising because IgE plays a pivotal role in allergic disease and the amount of IgE in the serum correlates with the disease severity^24, 25^. Recently, a humanized monoclonal antibody, quilizumab, was designed to neutralize IgE-expressing B cells, thereby depleting the net amount of IgE in the serum^26^. Although the therapy was able to reduce serum total and allergen-specific IgE by 30–40 %, it was not able to reduce the asthma exacerbations, lung function, or patient-reported symptoms^27^. Another murine humanized anti-IgE antibody, omalizumab, has been developed and is capable of binding IgE in the serum of allergic patients^28^. However, the administration of very high doses of omalizumab is required in patients because of the high sensitivity of mast cells and basophil granulocytes to IgE. Thus, the major drawbacks of this therapy include the high costs and the restriction of the therapy to a small group of patients with severe asthma^29^. Recently, a novel IgE-binding protein (DARPin E2_79) was developed. DARPin E2_79 is a so-called designed ankyrin repeat protein (DARPin), a small variant derived from a very broad class of naturally occurring ankyrin repeat proteins^30, 31^. Due to its high specificity to IgE, DARPin E2_79 is capable of inhibiting the formation of IgE–FcεRI receptor complexes^32^. At high concentrations, DARPin E2_79 has also been shown to disrupt previously formed complexes^33^ and thus prevents the onset of an allergic reaction.

Here, we report genetically engineered human cells able to produce IgE inhibitors at a precise time and in a dose-dependent manner to prevent the development of allergic inflammatory responses. The effective and accurate production of IgE inhibitors that is initiated by the two cytokines IL-4 and IL-13 dampens the histamine release in whole blood cell cultures. The use of such mammalian designer cells defines a major advance in the prevention, treatment and cure of beginning allergic reactions and helps alleviating allergic rashes, allergic symptoms and preventing daily administration of medications^34, 35^. Hence, the development of implantable cell-based synthetic cytokine sensor-effector devices may represent an important therapeutic advancement compared to conventional treatment strategies that may help to improve the quality of life of allergic patients.

## Results

### The design of the dual T_H_2 cytokine sensor device

**Fig. 1:**
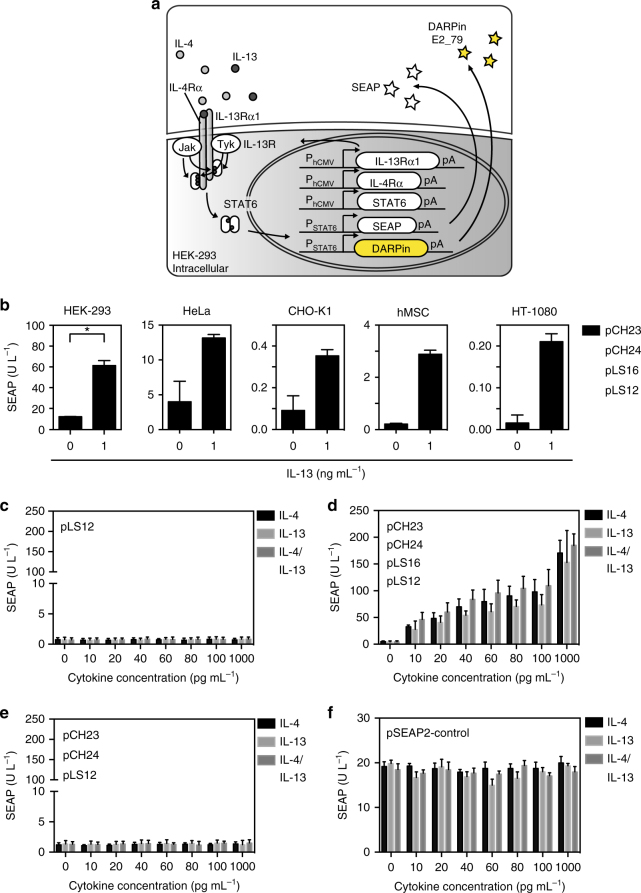
Design and validation of the dual T_H_2 cytokine sensor (DCS) device. **a** Schematic of the DCS. The two receptor subunits (IL-4Rα and IL-13Rα1) of the IL-13 receptor (IL-13R) and the transcription factor STAT6 are expressed constitutively. Activation of the IL-13 receptor by IL-4 and/or IL-13 results in the activation of the JAK/STAT pathway, and the subsequent phosphorylation of STAT6 drives its dimerization and translocation to the nucleus. The STAT6 dimers bind to a STAT6-specific operator, which is linked to a minimal promoter to control expression of either the SEAP or the IgE-binding protein DARPin E2_79. **b** Performance of the DCS device in different mammalian cell lines. HEK-293, HeLa, CHO-K1, hMSC, and HT-1080 were co-transfected with expression vectors encoding the IL-13Rα1 receptor subunit (pCH23, P_hCMV_-*IL13RA1*-pA), the IL-4Rα receptor subunit (pCH24, P_hCMV_-*IL4RA*-pA), the STAT6 transcription factor (pLS16, P_hCMV_-*STAT6*-pA) and the SEAP reporter gene (pLS12, P_STAT6/cEBP_-*SEAP*-pA). *p < 0.05, two-tailed Student’s *t*-test. **c** DCS performance in the absence of heterologous expression of IL-13 receptor components and STAT6. 3 × 10^4^ HEK-293 cells were transfected with the SEAP expression vector pLS12 (P_STAT6/cEBP_-*SEAP*-pA) and exposed for 48 h to different physiological cytokine concentrations (IL-4, IL-13, or both). **d** SEAP expression kinetics of the optimized DCS device in response to different cytokine concentrations. 3 × 10^4^ HEK-293 co-transfected with pCH23 (P_hCMV_-*IL13RA1*-pA) pCH24 (P_hCMV_-*IL4RA*-pA), pLS16 (P_hCMV_-*STAT6*-pA) and pLS12 (P_STAT6/cEBP_-*SEAP*-pA) were cultivated for 48 h in the presence of different physiological cytokine concentrations (IL-4, IL-13, or both). **e** STAT6 is an essential component for DCS activity. 3 × 10^4^ HEK-293 cells were co-transfected with pCH23 (P_hCMV_-*IL13RA1*-pA), pCH24 (P_hCMV_-*IL4RA*-pA) and pLS12 (P_STAT6/cEBP_-*SEAP*-pA), and cultivated for 48 h in the presence of different physiological cytokine concentrations (IL-4, IL-13, or both). **f** IL-4 and IL-13 have no impact on the viability and metabolic integrity of the DCS-transgenic cell line. 3 × 10^4^ HEK-293 cells were transfected with pSEAP2-Control (P_SV40_-*SEAP*-pA) and exposed for 48 h to different physiological cytokine concentrations (IL-4, IL-13, or both). SEAP levels were assessed after 48 h in the culture supernatant. The data represent the means±s.d. (*n* ≥ 3 independent experiments)

The design of the synthetic mammalian cell-based dual T_H_2 cytokine sensor (DCS) device is based on a common IL-13 receptor (IL-13R) that is shared by the allergy-associated cytokines IL-4 and IL-13 (Fig. 1a). The IL-13 receptor is composed of two subunits IL-4Rα and IL-13Rα1. The binding of IL-4 or IL-13 to their corresponding subunits leads to the recruitment of the opposite chains, either the IL-13Rα1 (for IL-4) or the IL-4Rα chain (for IL-13). The resulting dimerization of the two receptor subunits initiates a phosphorylation cascade involving members of Janus kinase/signal transducers and activators of transcription (JAK/STAT) pathway and the tyrosine kinase (TYK), resulting in STAT6 dimerization and subsequent localization to the nucleus. Gene expression is induced upon binding of the STAT6 dimer to its corresponding promoter consensus sequence (Fig. 1a).

A pre-optimized version of the DCS was tested in a set of different human and rodent cell lines (Fig. 1b, Supplementary Fig. 1). Human mesenchymal stem cells (hMSCs) showed the best fold induction of the system; however, human embryonic kidney cells (HEK-293) had the best overall performance with the highest SEAP gene expression rate. Thus, the different performances of the various cell lines may be explained by different endogenous expression levels of members of the JAK/STAT pathway necessary for the proper signal transduction of the IL-4 and IL-13 cytokine-sensing system^36–39^. For the DCS input, it has been shown that the IL-4 and IL-13 cytokines have short half-lives, and their concentration in the blood is very low (10^−12^ to 10^−10^ M)^40, 41^. To enable optimal signal detection and high sensitivity of the DCS, the co-transfection of the constructs pCH23 (P_hCMV_-*IL13RA1*-pA) and pCH24 (P_hCMV_-*IL4RA*-pA) encoding for the IL-13 receptor subunits combined with pLS16 (P_hCMV_-*STAT6*-pA) for the constitutive STAT6 gene expression was necessary (Fig. 1c–e). Additionally, the transfected reporter plasmid pLS12 (P_STAT6/cEBP_-*SEAP*-pA) enabled the controlled production and secretion of the human placental secreted alkaline phosphatase (SEAP) in response to input cytokines. After optimization of the plasmid ratios in HEK-293 cells, the step-by-step addition of individual DCS components showed considerable improvement in signal detection (Supplementary Fig. 2a–c). The HEK-293 cells only transfected with reporter plasmid pLS12 (P_STAT6/cEBP_-*SEAP*-pA) displayed very low leakiness (Fig. 1c). When transfecting STAT6-expressing plasmid pLS16 (P_hCMV_-*STAT6*-pA) and reporter plasmid pLS12 (P_STAT6/cEBP_-*SEAP*-pA), the SEAP activity in the presence of high input levels suggests that HEK-293 cells express the IL-13Rα1 and IL-4Rα receptor chains endogenously (Supplementary Fig. 2a). Additional co-transfection of either the IL-4Rα (pCH24) or IL-13Rα1 (pCH23) subunit resulted in increased gene expression and higher signal sensitivity of the DCS device (Supplementary Fig. 2b and c, respectively). However, only the co-transfection of both receptor subunits enabled increased and dose-dependent gene expression (Fig. 1d) with high fold induction: up to 41-fold for IL-4, 33-fold for IL-13 and 46-fold in the presence of both cytokines. The distinctive dose-dependent SEAP production in response to equal IL-4 or IL-13 concentrations could be explained by the heterodimeric IL-13 receptor complex kinetics in HEK-293 cells^42, 43^. Furthermore, we demonstrated that the receptor alone does not elicit any response in HEK-293 cells and that the transcription factor STAT6 (pLS16) is essential for the functionality of the DCS device (Fig. 1e). A mathematical model (Supplementary Fig. 2d) was developed for the analysis of the in vitro results of Fig. 1 and Supplementary Figs. 1 and 2. Resulting *p*-values indicate the significance of the correlation of the increasing cytokine concentration and the resulting SEAP units. As expected, the *p*-values show a high significance for Figs. 1c, d, and Supplementary Figure 2a–c, and are not significant in Figs. 1c, e, f, with *p* < 0.00001 (two-tailed Student’s *t*-test, Supplementary Fig. 2d). To test whether the device can be triggered by cytokines other than IL-4 and IL-13, a selection of different cytokines was added to the DCS device and it was shown that the IL-13R is specific and responds to IL-4 and IL-13 only (Supplementary Fig. 3a and b). No cytotoxicity of the cytokines was detected as constitutive expression of SEAP in the HEK-293 cells was not affected by any cytokine concentration used in this study (Fig. 1f).

### The DCS device rapidly and reversibly produces DARPin E2_79

**Fig. 2:**
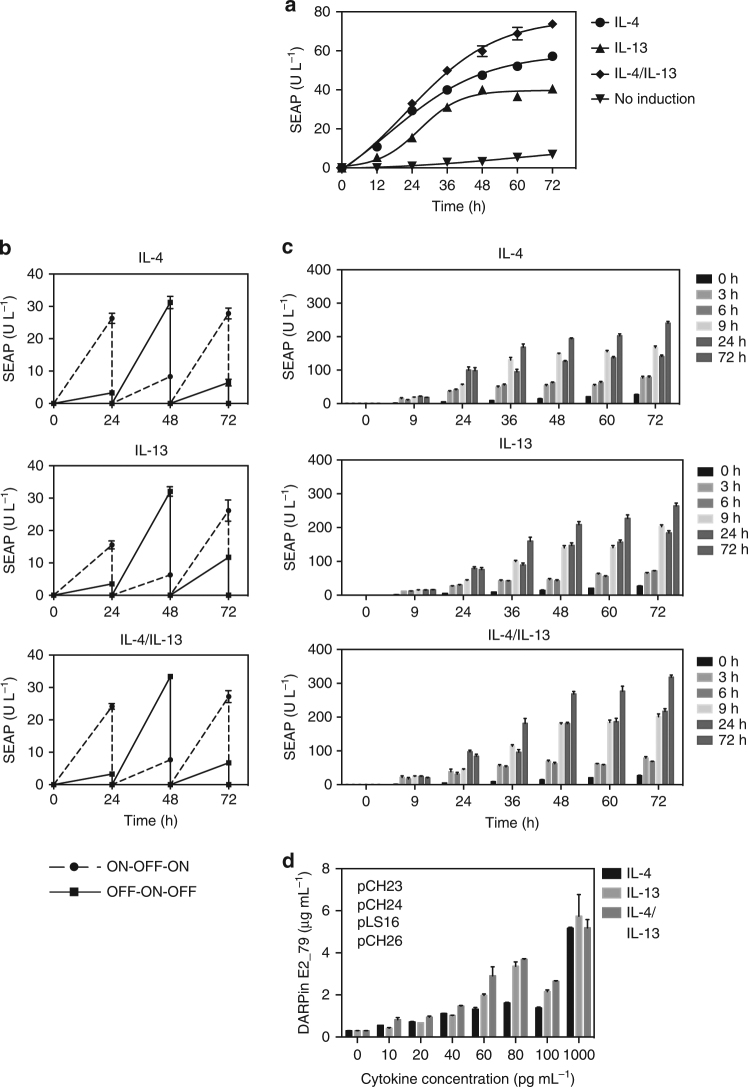
Characterization of DCS performance. **a** DCS induction kinetics. 3 × 10^4^ were co-transfected with pCH23 (P_hCMV_-*IL13RA1*-pA), pCH24 (P_hCMV_-*IL4RA*-pA), pLS16 (P_hCMV_-*STAT6*-pA) and pLS12 (P_STAT6/cEBP_-*SEAP*-pA) and exposed to different physiological cytokine concentrations (IL-4, 40 pg mL^−1^; IL-13, 80 pg mL^−1^; or both) for 72 h while SEAP levels were profiled in the culture supernatant at 12 h intervals. **b** Reversibility of the DCS device. DCS-transgenic cells shown in **a** were cultivated for 72 h while alternating the cytokine status (IL-4, 40 pg mL^−1^ ON; 0 pg mL^−1^ OFF; IL-13, 80 pg mL^−1^ ON, 0 pg mL^−1^ OFF; or both) of the culture every 24 h. **c** Cytokine exposure time-dependent DCS induction kinetics. DCS-transgenic cells shown in **a** were exposed for indicated periods of time to different physiological cytokine concentrations (IL-4, 40 pg mL^−1^; IL-13, 80 pg mL^−1^; or both) and SEAP expression was profiled in the culture supernatant at regular intervals. **d** DCS-driven DARPin E2_79 production in response to different cytokine concentrations. 3 × 10^4^ were co-transfected with pCH23 (P_hCMV_-*IL13RA1*-pA), pCH24 (P_hCMV_-*IL4RA*-pA), pLS16 (P_hCMV_-*STAT6*-pA) and pCH26 (P_STAT6/cEBP_-*SS*_*SEAP*_ *-His-DARPin E2_79*-pA) and exposed for 48 h to different physiological cytokine concentrations (IL-4, IL-13, or both) before DARPin E2_79 levels were profiled in the culture supernatant. The data represent the means±s.d. (*n* ≥ 3 independent experiments)

A series of experiments was performed to evaluate the kinetics of the DCS device. DCS-engineered cells exhibited a tightly regulated SEAP expression profile in the presence of pathophysiological levels of cytokines IL-4 and IL-13 in the low picomolar range over 72 h (Fig. 2a). Next, we demonstrated that the SEAP expression of the DCS device is reversible and can be turned on and off when alternating the different cytokine concentration in the culture medium (Fig. 2b). Transient exposure of cytokines to DCS-transgenic cells showed that a 3 h pulsing period is sufficient to activate the DCS device (Fig. 2c).

To program the DCS device for the treatment of allergic inflammatory responses, the SEAP reporter encoded by pLS12 (P_STAT6/cEBP_-*SEAP*-pA) was replaced by the IgE inhibitor DARPin E2_79. Initially, the sequence of the IgE-binding protein was optimized for its production in *Escherichia coli* ^32, 33^. The sequence of the protein used for this work has therefore been codon-optimized for human protein translation and has been equipped with a human secretion signal (SS_SEAP_) to ensure its correct production and secretion by mammalian cells, respectively. The DARPin E2_79-expressing construct pCH26 (P_STAT6/cEBP_-*SS*_*SEAP*_ *-His-DARPin E2_79*-pA) was cloned by replacing the SEAP gene of pLS12 (P_STAT6/cEBP_-*SEAP*-pA) with the human codon-optimized and secretion-engineered DARPin E2_79 sequence. As expected, the DCS-transgenic cells secreted the IgE inhibitor protein DARPin E2_79 in a dose-dependent manner into the supernatant of engineered cells when exposed to varying concentrations of IL-4 and/or IL-13 (Fig. 2d).

As the DCS device is designed to be used in the whole blood of allergic donors, HEK-293 cells engineered for DCS-controlled DARPin E2_79 production were microencapsulated in alginate-poly-(*L*)-lysine-alginate capsules to protect the cells from the attack of human immune cells contained in the whole blood. To ensure the correct performance of the encapsulated cells, the dose-dependent DARPin E2_79 expression (Supplementary Fig. 4a and b) and the expression kinetics (Supplementary Fig. 4c and b) of the DCS device and the controls were profiled in vitro. The alginate-poly-(l)-lysine-alginate capsules were shown here to be permeable for the cytokines IL-4 and IL-13, both necessary to trigger the DARPin E2_79 production of the encapsulated cells. Furthermore, the dose-dependent DARPin E2_79 expression (Supplementary Fig. 4e and 4f) and the expression kinetics (Supplementary Fig. 4g and 4h) of the DCS device were profiled ex vivo using the blood of a non-allergic donor to ensure the correct functioning of the DCS device in the presence of human whole blood.

### Cell-produced DARPin E2_79 disrupts the IgE-FcεRI complex

Because the high-affinity interaction of DARPin E2_79 with IgE is critical to hinder the formation of a stable IgE-FcεRI receptor complex, it was essential to show that the DARPin E2_79 produced in mammalian cells has similar binding properties as the reported *E. coli*-derived DARPin E2_79. Successful binding of the mammalian DARPin E2_79 to human IgE and disruption of the IgE-FcεRI receptor complex was shown by fluorescence quenching experiments. Increasing concentrations of DARPin E2_79 produced in mammalian HEK-293 cells were incubated with human IgE and subsequently added to the immobilized recombinant human FcεRI receptor. Increasing concentrations of DARPin E2_79 resulted in the complete prevention of the IgE-FcεRI receptor complex formation in vitro (Supplementary Fig. 5a). A 10-fold excess of the DARPin E2_79 concentration toward IgE was sufficient to hinder the binding of nearly all IgE present in the sample. A second in vitro experiment showed that increasing concentrations of mammalian cell-produced DARPin E2_79 enabled the disruption of previously formed IgE-FcεRI receptor complexes, which indicated that the binding and disrupting properties of the DARPin E2_79 are not altered when produced in mammalian cells (Supplementary Fig. 5b). These results are consistent with previous reports, where similar experiments using DARPin E2_79 produced in *E. coli* have shown that its affinity is in the nM range (*K*_D_ 6.29 × 10^−9^M)^33^. Also, the half-maximal disruptive concentration of the IgE-FcεRI complexes shows that µM concentrations are required to disrupt the IgE-receptor interaction (ID_50_ 1.57 × 10^−6^M)^44^.

**Fig. 3:**
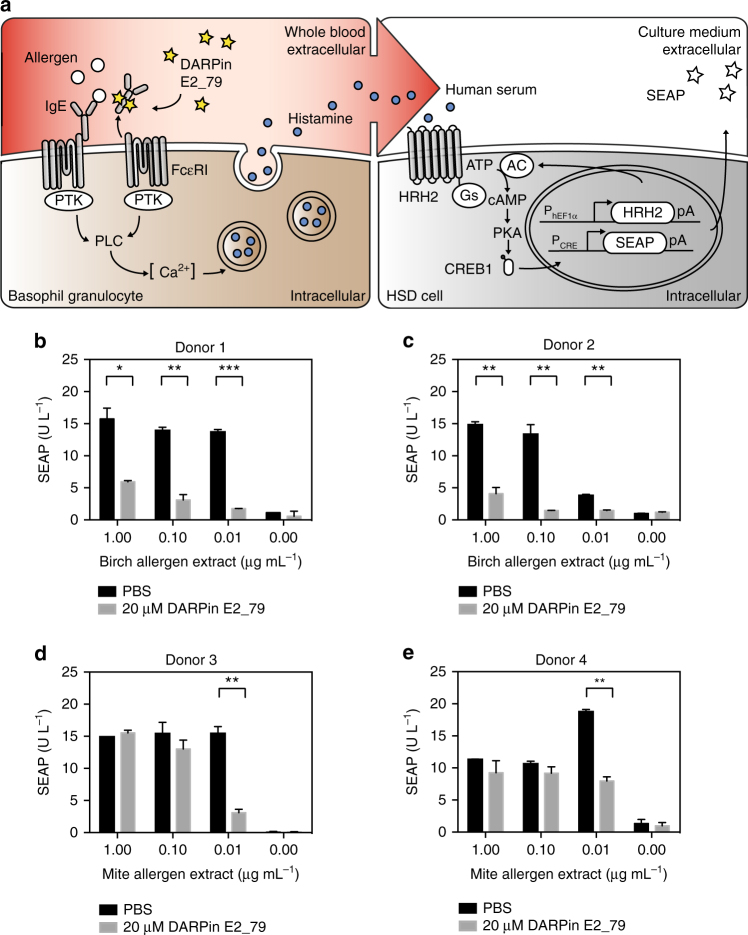
Functional characterization of DARPin E2_79 produced in mammalian cells. **a** Illustration of the operational procedure for the analysis of mammalian cell-produced DARPin E2_79. The basophil granulocytes in donor blood samples exposed to the allergen as well as DARPin E2_79 activate their FcεRI receptor and trigger the underlying signaling cascade involving protein tyrosine kinase (PTK) and phospholipase C (PLC) which results in a calcium (Ca^2+^) surge and corresponding release of histamine, which can be quantified using reporter cells containing a histamine sensor device (HSD cells). The HSD-transgenic cells detect histamine via the human histamine receptor H2 (HRH2) which is rewired to a synthetic signaling cascade involving G-protein s (G_s_), adenylate cyclase (AC), cyclic AMP (cAMP), protein kinase A (PKA) and the cAMP-responsive binding protein 1 (CREB1) to activate SEAP from a P_CRE_-driven promoter. SEAP levels correlate with the allergen-triggered histamine release and provide quantitative information on DARPin E2_79’s capacity to interfere with a patient-specific allergic response. **b**–**e** DARPin E2_79-mediated disruption of the IgE–FcεRI receptor complex in the whole blood of four donors allergic to **b**, **c** birch and **d**, **e** house dust mite allergens using the whole-blood culture system shown in **a**. The human blood samples of the four donors were each supplemented with 20 µM mammalian cell-produced DARPin E2_79 3 h prior to the addition of different concentrations of the respective allergens. The resulting histamine release was analysed using 5 × 10^4^ HSD-transgenic HEK-293 cells and the SEAP levels were profiled after 24 h. Data are means±s.d. of each blood donor sample measured in duplicate. *p < 0.05, **p < 0.005, ***p < 0.0001, two-tailed Student’s *t*-test

Similar to these findings, the IgE disrupting proteins were found to be equally effective in the blood of allergic subjects. DARPin E2_79 produced in FreeStyle™ 293-F serum-free suspension cultures and incubated with whole-blood samples of allergic donors was able to drive the disruption of the IgE–FcεRI receptor complexes present on the surface of basophil granulocytes (Fig. 3a). In detail, different concentrations of allergen extracts were added to the whole blood to trigger histamine release by basophil granulocytes. Subsequent addition of this histamine-containing serum to the histamine sensor-transgenic designer cells^45^ enabled the quantification of released histamine via the production of SEAP (Fig. 3a). The addition of 20 µM DARPin E2_79 to the whole blood of two blood donors allergic to birch pollen, 3 h prior to the addition of birch pollen extract, nearly eliminated the entire histamine release of the basophil granulocytes as assessed by the reduced production of SEAP by the histamine sensor cells (Figs. 3b and c). In the blood of a donor allergic to the house dust mite, the histamine release in the presence of 20 µM DARPin E2_79 was reduced by almost 95% at 0.01 µg mL^−1^ allergen concentrations (Fig. 3d) while it stayed constant for another donor allergic to the house dust mite, although there was a considerable increase in the histamine release at 0.01 µg mL^−1^ allergen concentrations, resulting in almost 58% less histamine (Fig. 3e). The different patterns of allergen stimulation are due to non-ideal allergen responses curves of the blood basophil granulocytes and have been described elsewhere^45, 46^. DARPin E2_79 has been well characterized in vitro as was its capacity to dissociate the IgE from its receptor without allergen challenge^47^. In order to specifically address the therapeutic potential of DARPin E2_79 produced by the DCS, a quantification of the dissociation of the IgE-FcεRI complex in human whole blood in the absence of allergen using flow cytometry was performed, showing that DARPin E2_79 of mammalian origin was able to disrupt the IgE from its receptor, thereby showing a dose-dependent reduction in the fluorescence signal (Supplementary Fig. 6a–c). A trypan blue-based viability assay of HEK-293 cells transfected with the DCS device exposed to increasing concentrations of DARPin E2_79 did not reveal cytotoxicity of any DARPin E2_79 concentration used in this study (Supplementary Fig. 7).

### DCS detects cytokines in blood and secretes DARPin E2_79

**Fig. 4:**
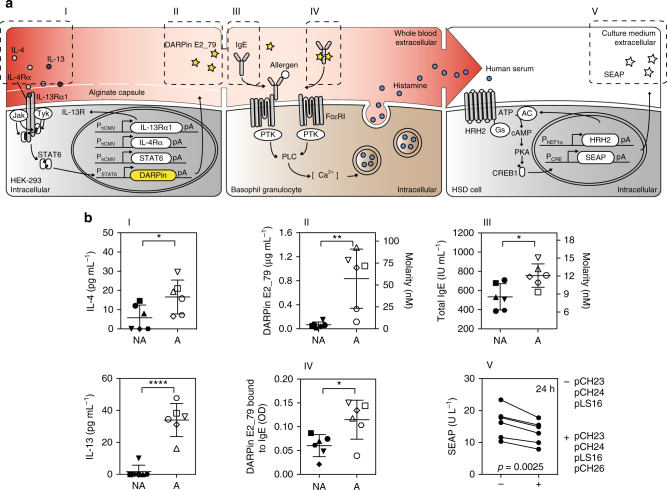
DCS-controlled DARPin E2_79 production in the human blood. **a** Illustration of the operational analysis procedure of the DCS-controlled DARPin E2_79 performance in human whole blood. 8 × 10^5^ microencapsulated HEK-293 cells transgenic for DCS-controlled DARPin E2_79 (200 cells per capsule, 4000 capsules) were cultivated in 0.5 mL of human blood diluted 1:1 in RPMI-1640 for 24 h during which the allergen-associated cytokines IL-4 and IL-13 in the donor’s blood (I) trigger DCS-controlled production and secretion of DARPin E2_79 (II). IgE in the blood of the different donors (III) is bound by the produced DARPin E2_79 (IV). Addition of allergen extracts results in specific release of histamine by the basophil granulocytes in the donor’s blood. Histamine levels and thus overall DCS performance is quantified by histamine-sensitive SEAP production (V) using a HSD-transgenic reporter cell line (HSD cells). **b** Quantitative analysis of the DCS performance parameters including (I) IL-4 and IL-13 concentrations (24 h), (II) DARPin E2_79 production levels (24 h), (III) total IgE levels (24 h) and (IV) DARPin E2_79-bound IgE in the blood of non-allergic (NA) and allergic (A) donors (24 h). Symbols are assigned to each donor throughout graphs I, II, III, and IV. (V) Allergen-triggered DCS-controlled DARPin E2_79 impact on histamine release **a**, **b**. Allergen extract was added to the whole-blood sample of six allergic donors that contained the microencapsulated HEK-293 cells transgenic for DCS-controlled DARPin E2_79 production (**a**, **b**, V). Control cells lacking DCS’s DARPin E2_79 reporter gene pCH26 (P_STAT6/cEBP_-*SS*_*SEAP*_ *-His-DARPin E2_79*-pA) were run in parallel. Whereas histamine levels remain high in the control group, the histamine release of the blood basophil granulocytes is reduced significantly to basal levels after 24 h. Data represent means ± s.d. of two histamine release assays. I, II, III, IV: Data are means±s.d. of each blood donor sample (*n* = 6) measured in duplicate. *p < 0.05, **p < 0.005, ****p < 0.00001, two-tailed Student’s *t*-test. V: Data are means±s.d. of each blood donor sample (*n* = 6) measured in duplicate. Paired *t*-test: *t*(5) = 5.6, *p* = 0.0025

Next, it was essential to show whether DCS-transgenic cells were able to detect cytokines in the blood of allergic donors and, in response, fine tune the secretion of DARPin E2_79. Therefore, HEK-293 cells engineered for DCS-controlled DARPin E2_79 production were microencapsulated in alginate-poly-(l)-lysine-alginate capsules (Fig. 4a). Six allergic blood donors with positive skin prick tests and six non-allergic donors were selected for the experiments. The various allergy parameters IL-4 and IL-13, DARPin E2_79, the total IgE contained in the serum of the donors and the IgE-DARPin E2_79 complexes were monitored by ELISA (Fig. 4b, I-IV). The cytokine levels of IL-4 have previously been shown to correlate with the IgE concentrations in the blood of allergic donors^48^. As expected, upon co-cultivation of microencapsulated cells transgenic for DCS-controlled DARPin E2_79 expression in the whole-blood samples of the allergic subjects, the increased cytokine concentrations in the donors’ blood triggered substantial production of DARPin E2_79, which exceeded 0.8 µg mL^−1^ in the plasma after 24 h (Fig. 4b, II). In contrast, the low levels of cytokines present in the blood of the non-allergic donors resulted only in basal expression of DARPin E2_79 (up to 0.08 µg mL^−1^). The cytokine concentrations correlated with DARPin E2_79 production and total IgE levels. The cytokine and total IgE levels remained also low for the non-allergic group. Calculations of the DARPin E2_79 over IgE ratios (Supplementary Fig. 8) show that the molar excess of IgE inhibitor over IgE for the majority of the donors ranges from 5.2-fold up to 7.7-fold. These concentrations prevented the formation of more than 50% of IgE-FcεRI receptor complexes (5-fold molar excess) and up to 100% of IgE-FcεRI receptor complexes (10-fold molar excess), respectively (Supplementary Fig. 5a). Finally, it was shown that IgE and DARPin E2_79 formed complexes in the donor serum, thereby showing that the IgE inhibitors produced by mammalian cells were effectively binding the IgE present in the plasma of the blood donors (Fig. 4b, IV). A control experiment consisting of constitutive expression of the DARPin E2_79 (pCH15, P_hCMV_-*SS*_*SEAP*_ *-His-DARPin E2_79*-pA), reduced the histamine release of the basophil granulocytes significantly (Supplementary 9a–f).

To test whether the DCS device produced sufficient amounts of DARPin E2_79 to reduce histamine levels during an allergic response, the microencapsulated DCS-responsive DARPin E2_79 producer cells were maintained for 24 h (Fig. 4b, V, and Supplementary Fig. 10a) and 72 h (Supplementary Fig. 10b and c) in the blood of six allergic donors. A control consisting of microencapsulated HEK-293 cells engineered for constitutive expression of the IL-13 (pCH23, P_hCMV_-*IL13RA1*-pA) and IL-4 receptors (pCH24, P_hCMV_-*IL4RA*-pA) as well as STAT6 (pLS16, P_hCMV_-*STAT6*-pA) was run in parallel in the whole-blood samples of the same donors. The addition of allergen extract triggered histamine release by basophil granulocytes (Fig. 4b, V). The produced DARPin E2_79 reduced the histamine release at 24 h (Fig. 4b, V) and at 72 h (Supplementary Fig. 10b and c) for all blood donors, compared to the control blood samples, which suggests that the DCS-controlled DARPin E2_79 production could indeed significantly reduce the release of histamine. The release of histamine in the presence or absence of allergen is shown in Supplementary Fig. 10a and c to rule out a premature loss of functionality of the basophil granulocytes after 24 or 72 h in the whole blood cell culture.

Next, an improved variant of the encapsulated DCS device with increased DARPin E2_79 expression levels and slightly higher basal levels (Supplementary Fig. 11) was used for the assessment of histamine release by the validated reporter cell line and for the direct evaluation of basophil activation by a basophil activation test (BAT). The cells were encapsulated and added to the blood of six allergic donors for 24 h (Supplementary Fig. 12). As expected, the histamine release was significantly reduced in the presence of the DARPin E2_79 producing DCS. A basophil activation test run in parallel showed also a significant reduction of the CD63 positive basophil granulocytes (Supplementary Fig. 12) for the six allergic donors. Higher amounts of DARPin E2_79 were produced by the optimized version of the DCS device (maximal DARPin E2_79 concentrations ranged between 1.3 and 2 µg mL^−1^) although the cytokines levels in the blood samples where somewhat lower than in Fig. 4b, I, where the cytokine concentrations caused maximal DARPin E2_79 concentrations between 1 and 1.3 µg mL^−1^, showing that the optimized DCS device was able to make up for the lower cytokine concentration in the blood. Furthermore, the DARPin E2_79 concentrations produced by the improved variant of the DCS device were enough to abolish the histamine release for blood donors no. 4, 5, and 6, after 24 h (Supplementary Fig. 12b), indicating a clear improvement compared to the previous DCS device (Supplementary Fig. 10a).

The dual cytokine sensor device presented here enables the precise monitoring of the cytokines and allergic markers IL-4 and IL-13 in the blood of allergic donors. Due to a rewired synthetic pathway of the sensor device, the cytokines drive the production of a novel IgE-binding protein, DARPin E2_79, thereby intercepting the IgE in the blood. Previous work on IgE-binding molecules have shown that basophil granulocytes provide an established readout to characterize the properties of IgE-inhibiting proteins ex vivo^44, 47^. Using these cells, it was shown that the DCS device leads to a reduction of the histamine release upon allergen trigger by the basophil granulocytes.

## Discussion

Synthetic biology has significantly advanced the design of trigger-inducible genetic sensor switches that can be assembled to complex sensor-effector devices known as prosthetic gene networks^49^. Prosthetic gene networks coordinate disease detection to therapeutic intervention using Boolean processing logic and closed-loop control capacity^50–54^. Implantable designer cells containing prosthetic networks represent a fundamental step forward for personalized medicine because they link diagnosis to treatment and provide precise on-demand in situ production, dosing and systemic delivery of protein pharmaceuticals^55^. Here, we provide a synthetic biology-inspired designer cell-based strategy for the treatment of allergies and asthmatic conditions. Both are recurrent medical conditions and especially allergic asthma can be persistent^56^. Typical symptoms of asthma consist in recurrent dry coughing, wheezing, chest tightness, or shortness of breath, and often these symptoms can be severe or life-threatening^57, 58^. Especially because of the severity of the symptoms, the patients are forced to take daily medications and frequently, these anti-allergic medications have considerable side effects such as drowsiness, dizziness and nausea^59, 60^. Intercepting IgE in the blood of allergic and asthmatic patients has been shown to be fundamental^56, 61^, hence, rewired designer cells optimized for the production of novel IgE-inhibiting proteins allow to reliably protect from allergic asthmatic symptoms and relieve the patients from life-threatening sentiments. Cell-based therapies which couple real-time biomarker sensing to in situ production of protein therapeutics such as the pioneering DCS device reported here, provide a novel level of treatment dynamics that cannot be achieved by injection-based administration of protein pharmaceuticals^62^. Cells can sense their surroundings and are able to react in complex and sophisticated ways, and as therapeutical devices solve thereby the challenges of the precise therapeutic dosing and optimal times of administration^34, 63, 64^. In this regard, the DCS device is proposed as novel tool and is part of the important development of biomedical science^45^.

The presented DCS device has been optimized to detect the onset of an allergic response and to sense the presence of IL-4 and IL-13 cytokines during the allergic disease. Upon exposure of the immune system to specific allergens, T_H_2 cells release high amounts of cytokines, mainly IL-4 and IL-13. Both signals can activate the DCS device either individually or in combination, which guarantees a high sensitivity and a broad signal-sensing capacity. These allergy-associated cytokines trigger the DCS device to quickly produce the synthetic protein DARPin E2_79, which is capable of binding human IgE. The steady and increasing production of IgE inhibitors by the implanted cells is meant to quickly intercept the IgE released by the plasma cells. Because IL-4 drives IgE production, it was demonstrated that high amounts of this cytokine correlates with high amounts of IgE in the blood of patients^65, 66^. As shown in vitro (Figs. 1d and 2d), the DCS device has been engineered to sense minute amounts of cytokines and to coordinate the production of IgE-inhibiting DARPin E2_79: the higher the cytokine levels—the higher the DARPin E2_79 production. Since the cytokine levels in allergic individuals correlate with IgE levels^19, 66^, the engineered human cells continuously monitor allergy-related cytokine levels, detect the increased demand for DARPin E2_79 and ramp up DARPin E2_79 production and secretion accordingly. Fluctuations in allergen concentrations (due to seasonal changes or irregular allergen exposure) are perceived by the DCS device, thereby adjusting the DARPin E2_79 concentration. Usage of the DCS device during a season with high pollen occurrence due to warm and dry temperatures (Fig. 4) and a season with lower pollen occurrence (due to unfavorable weather conditions, Supplementary Fig. 12) shows that the DCS device adequately drives the production of IgE-interfering molecules throughout the seasons. The synthetic DARPin E2_79 produced in *E*. *coli* has been well characterized in vitro and our synthetic biology-inspired cell-based therapy concept using a cytokine sensor/effector gene circuit capitalizes DARPin-mediated IgE and IgE-FcεRI targeting^32, 33, 44^.

This study shows that the DCS device fine-tuned its response to the allergy markers. Compared to our in vitro data, the concentrations of DARPin E2_79 over IgE would have been high enough to prevent formation of at least 50% of the IgE-FcεRI receptor complexes after only 24 h exposure of the DCS device to the allergy markers. Furthermore, the synthetic mammalian cell-based dual T_H_2 cytokine sensor device is based on engineered human cells that simultaneously detect the allergen-specific cytokines IL-4 and IL13 and correspondingly produce and secrete DARPin E2_79 to attenuate the allergic reaction by dampening associated histamine release. Both, the simultaneous detection of cytokines and the reduction of histamine release, have been achieved with the help of a synthetic mammalian cell device, this being a completely novel achievement for synthetic biology. The therapeutic vision of the DCS device is the in situ production of DARPin E2_79 in human cells in response to IL-4 and IL-13, thereby sequestering the IgE in the blood of allergic patients and inhibiting the formation of IgE–FcεRI complexes. The resulting reduction of free allergen-specific IgE is expected to decrease the sensitivity of patients toward allergens. We show here that the engineered human cells were able to produce the IgE-inhibiting molecule DARPin E2_79 in a 5-fold molar excess over IgE, a ratio that is sufficient to prevent the binding of almost 50% of IgE in vitro.

DCS-containing designer cells may develop into an alternative to established drug-based treatment of allergies. However, there are major challenges toward clinical translation. For example, the DCS device is difficult to validate in an in vivo context, the precision of the sense-and-response dynamics may turn out to be suboptimal and the treatment of acute manifestations of the allergic disease will be complicated. Nevertheless, the cell-based DCS strategy holds the promise for life-long protection of patients, in particular those with a genetic predisposition for allergies (e.g., atopic dermatitis^67^, Netherton’s disease^68^ or asthma-susceptible genes^69^). For the treatment of such disorders, the DCS device should ideally be implanted prior or during the sensitization phase to prevent the onset of an allergy. This may also prevent the development of late allergic phases of the disease and decrease the risk of recurrent allergic inflammations becoming chronic.

Human whole blood from allergic and non-allergic blood donors was chosen to assess the performance and response dynamics of the DCS device to allergic inflammatory responses. Mouse model of experimental allergy that enables a thorough characterization of the DCS device in vivo do not exist. Human FcεRI-transgenic mice have been generated^70^, but these animals do not produce the human IgE required to test the DARPin E2_79 in vivo. Therefore, the animals would need to be infused with human IgE or serum of allergic patients and - since they also lack the trigger cytokines—they would also need to receive injections of IL-4 and IL-13 as well, which makes this a poor and prohibitively artificial animal model.

As stated in the literature, the IgE-binding domains are efficiently blocked by the DARPin E2_79 molecules^33^. Once formed, the IgE-DARPin E2_79 complexes are extremely stable and have a very slow dissociation rate^47^. The presence of IgE inhibitors reduces the amount of free IgE in the blood^28^. FcεRI surface levels in mast cells and basophil granulocytes decrease as the serum IgE concentration decreases in humans and mice^71, 72^—this effect, which likely reduces the ability of mast cells and basophil granulocytes to sense and react to allergens during allergic responses^73^, was observed in several clinical studies with patients treated with Omalizumab^74, 75^. Patients with allergies are often prone to the development of additional allergies or affected by temporary allergies such that they turn into chronic inflammation^76^. Thus, the DCS may optimally prevent the establishment of additional allergies due to the synchronized interception of free IgE by DARPins. High cytokine amounts caused by a temporary allergy would increase the amount of IgE inhibitors in the blood due to the fine-tuned response of a cell implant, thereby reducing the total amount of free IgE. Since it has been shown that the loss of free FcεRI ligands reduces the net amount of FcεRI, a cell-based device could greatly diminish the risk for chronic allergies. Also, the DCS device has been shown to be responsive to cytokines in human mesenchymal stem cells. It has been described that human induced pluripotent stem cells (hIPSCs)-derived pancreatic progenitor cells could be reprogrammed into glucose-sensitive insulin-secreting beta-like cells^63^. Similarly, this provides the unique opportunity of rewiring patient-derived stem cells to achieve personalized implants capable of protecting allergic patients reliably and durably. As the DCS device is programmed to interfere with the onset of perennial allergic and asthmatic reactions as well as attenuate ongoing allergic reactions, it is important to note that the treatment of other serious medical conditions such as life-threatening anaphylactic reactions are beyond the scope of the DCS device.

DARPin technology is relatively recent, and the construction of new generations of DARPins is very promising, bearing a vast development potential for IgE-binding DARPins^77, 78^. We show here for the first time that DARPin E2_79 can be successfully produced in mammalian cells without loss of functionality. Besides, due to their production in mammalian cells, the molecules are free of toxic contaminants such as bacterial endotoxins and are particularly safe for the use in patients. Using a biparatopic IgE-targeting approach, the disruptive potency of DARPin E2_79 has been improved by approximately 100-fold^47^. The production of novel generations of IgE-binding DARPins is very encouraging, especially if they are used in a precisely controlled manner.

## Methods

### Components of the DCS

Comprehensive design and construction details for all expression vectors are provided in Supplementary Table 1. The key components of the DCS device include pCH23 (P_hCMV_-*IL13RA1*-pA) and pCH24 (P_hCMV_-*IL4RA*-pA) for constitutive expression of the IL-13 receptor subunits, pLS16 (P_hCMV_-*STAT6*-pA), encoding constitutive expression of STAT6 to enable the synthetic signaling cascade and pCH26 (P_STAT6/cEBP_-*SS*_*SEAP*_ *-His-DARPin E2_79*-pA containing a human codon-optimized, polyhistidine-tagged (His) and secretion-engineered (SS_SEAP_) DARPin E2_79.

### Cell culture and transfection

Human embryonic kidney cells (HEK-293, ATCC: CRL-11268), human cervical carcinoma cells (HeLa, ATCC: CCL-2), immortalized human mesenchymal stem cells (hMSC; hMSC-TERT^79^) and human fibrosarcoma cells (HT-1080, ATCC: CCL-121) were cultured in Dulbecco’s modified Eagle’s medium (cat. no. 52100-039; DMEM, Life Technologies, Carlsbad, CA, USA) supplemented with 10% FCS (cat. no. 2-01F10-I; lot no. PE01026P; FCS, BioConcept, Allschwil, Switzerland) and a 1% penicillin/streptomycin solution (cat. no. P4333; Sigma-Aldrich, Munich, Germany). Chinese hamster ovary cells (CHO-K1, ATCC: CCL-61) were cultivated in ChoMaster HTS (cat. no. CHTS-8; Cell Culture Technologies, Gravesano, Switzerland) supplemented with 5% FCS and a 1% penicillin/streptomycin solution. FreeStyle 293-F cells (cat. no. R790-07; Life Technologies, Invitrogen, Carlsbad, CA, USA) were cultured in FreeStyle™ 293 Expression Medium (cat. no. 12338018; Life Technologies, Invitrogen, Carlsbad, USA). All of the cell types were cultured at 37 °C in a humidified atmosphere containing 5% CO_2_. The cell numbers and viability were quantified using an electric field multichannel cell-counting device (CASY Cell Counter and Analyzer Model TT; Roche Diagnostics GmbH, Rotkreuz, Switzerland). The cells were transfected using an optimized PEI transfection protocol. The cells were seeded 16 h before transfection at 3 × 10^4^ per well of a 48-well plate. The transfection was performed upon the addition of 0.4 µg plasmid DNA and 1.2 µL PEI (1 mg mL^−1^ stock solution, cat. no. 24765-2; Polyethylenimine “Max”, Polysciences Inc., Warrington, PA, USA) per well to FCS-free DMEM for 15 min at 22 °C before it was added dropwise to the cells. For the SEAP output, 0.0125 µg of pCH23 (P_hCMV_-*IL13RA1*-pA) and pCH24 (P_hCMV_-*IL4RA*-pA), 0.150 µg of pLS12 (P_STAT6/cEBP_-*SEAP*-pA) and 0.05 µg of pLS16 (P_hCMV_-*STAT6*-pA) were used. For the pre-optimized version of the DCS tested in a set of different human and rodent cell lines with SEAP output, 0.05 µg of pCH23 (P_hCMV_-*IL13RA1*-pA) and pCH24 (P_hCMV_-*IL4RA*-pA), 0.150 µg of pLS12 (P_STAT6/cEBP_-*SEAP*-pA) and 0.05 µg of pLS16 (P_hCMV_-*STAT6*-pA) were used. For regulated DARPin E2_79 production, 0.0125 µg of both pCH23 (P_hCMV_-*IL13RA1*-pA) and pCH24 (P_hCMV_-*IL4RA*-pA), 0.05 µg of pCH26 (P_STAT6/cEBP_-SS_SEAP_-His-Tag-DARPin E2_79-pA) and 0.05 µg of pLS16 (P_hCMV_-*STAT6*-pA) were used. For the increased expression of DARPin E2_79 with increased basal levels, 0.025 µg of both pCH23 (P_hCMV_-*IL13RA1*-pA) and pCH24 (P_hCMV_-*IL4RA*-pA), 0.1 µg of pCH26 (P_STAT6/cEBP_-*SS*_*SEAP*_ *-His-DARPin E2_79*-pA) and 0.1 µg of pLS16 (P_hCMV_-*STAT6*-pA) were used. After 6 h, the medium was replaced by either standard cultivation medium or medium supplemented with the indicated amounts of recombinant proteins, including human recombinant interleukin 4 (cat. no. 200-04; IL-4, PeproTech, Rocky Hill, NJ, USA) and human recombinant interleukin 13 (cat. no. 200-13; IL-13, PeproTech). Unless stated otherwise, the transgene expression was profiled after 48 h. For the expression and secretion of DARPin E2_79 in FreeStyle 293-F cells, 2 µg mL^−1^ of pCH15 and 6 µL of PEI per 1 × 10^6^ cells were mixed with FreeStyle 293 Expression Medium for 15 min at 22 °C before it was added to the cells. The cell culture supernatant was collected after 4 days.

### Analytical assays

SEAP levels: The production of human placental secreted alkaline phosphatase was quantified in the cell culture supernatant as described previously^80^. In brief, 100 µL of the cell culture supernatant was heat inactivated for 30 min at 65 °C. Subsequently, 80 µL of the supernatant was transferred to a well in a 96-well plate containing 100 µL 2× SEAP assay buffer (20 mM homoarginine, 1 mM MgCl_2_, 21% (v/v) diethanolamine, pH 9.8). After the addition of 20 µL of 120 mM para-nitrophenylphosphate (pNPP disodium salt, hexahydrate, cat. no. AC128860100; Acros Organics BVBA, Geel, Belgium) diluted in 1x SEAP assay buffer, the time-dependent increase in light absorbance was profiled at 405 nm for 30 min using a GeniosPro multi-well reader (Tecan, Maennedorf, Switzerland). DARPin E2_79 profiling: To assess the production of DARPin E2_79, human IgE (cat. no. 250203; Abbiotech, San Diego, CA, USA) was immobilized at 3 µg mL^−1^ on an ELISA plate (cat. no. CLS3590; Sigma-Aldrich, Munich, Germany). A mouse anti-His-tag secondary antibody (cat. no. 70796; Merck, Darmstadt, Germany; dilution 1 : 1000) was added to bind DARPin E2_79. A third sheep anti-mouse HRP-tagged antibody (cat. no. RPN4201; GE Healthcare, Chalfont St Giles, UK; dilution 1 : 1000) was used to detect DARPin E2_79. Cytokine measurement: The IL-4 and IL-13 levels in the blood samples were assessed using an IL-4- and IL-13-specific ELISA (IL-4, cat. no. 3410-1H-6; IL-13, cat. no. 3710-1H-6; Mabtech, Nacka Strand, Sweden) according to the manufacturer’s instructions. IgE quantification: To assess the total IgE level in the serum, goat α-human IgE (cat. no. AS10749; Agrisera, Vännäs, Sweden) was immobilized at 2.5 µg mL^−1^ overnight at 4 °C on an ELISA plate (cat. no. CLS3590; Sigma-Aldrich, Munich, Germany). The serum was diluted 1:1 with Mabtech Ready-to-use diluent for ELISA (cat. no. 3652-D2; Mabtech, Nacka Strand, Sweden). An anti-human Ig kappa light-chain FITC (cat. no. 11-9970-42; Affymetrix eBioscience, San Diego, CA, USA; dilution 1 : 200) was then added to detect the bound IgE. Quantification of IgE-DARPin E2_79 complexes: To assess the IgE-DARPin E2_79 complexes in the serum, goat anti-human IgE (cat. no. AS10749; Agrisera, Vännäs, Sweden) was immobilized at 5 µg mL^−1^ overnight at 4 °C on an ELISA plate (cat. no. CLS3590; Sigma-Aldrich, Munich, Germany). A mouse anti-His-tag secondary antibody (cat. no. 70796; Merck, Darmstadt, Germany; dilution 1 : 1000) was added to bind DARPin E2_79. A third sheep anti-mouse HRP-tagged antibody (cat. no. RPN4201; GE Healthcare, Chalfont St Giles, UK; dilution 1 : 1000) was used to detect DARPin E2_79. Histamine release from human basophil granulocytes: The release of histamine from human basophil granulocytes was quantified as previously described^45^. In brief, 200 µL of whole blood containing the microencapsulated DCS device were mixed with the indicated concentrations of allergen with an adapted protocol using DARPin E2_79, anti-human IgE, the allergens and fMLF diluted in PBS instead of degranulation buffer. The histamine release assay was performed in an incubator at 37 °C in a humidified 5% CO2-containing atmosphere for 30 min. Thereafter, the cells were centrifuged for 10 min at 700 × *g* at 4 °C. The resulting serum was either directly used for subsequent assays or was stored at −20 °C.

### Purification of DARPin E2_79

The purification of His-tagged DARPin E2_79 was performed under native conditions. In brief, a cell culture supernatant of FreeStyle 293-F cells transfected with the DARPin E2_79 expression vector pCH15 (P_hCMV_-*SS*_*SEAP*_ *-His-DARPin E2_79*-pA) was incubated overnight at 4 °C with equilibrated Ni-NTA agarose (cat. no. 745400.25; Macherey Nagel, Düren, Germany). After centrifugation of the agarose, the supernatant was discarded and washing steps were performed with NPI-20 wash buffer (50 mM NaH_2_PO_4_; 300 mM NaCl; 10 mM imidazole; pH 8). The elution of DARPin E2_79 was performed using NPI-250 elution buffer (50 mM NaH_2_PO_4_; 300 mM NaCl; 250 mM imidazole; pH 8). Finally, the DARPin E2_79 fraction was concentrated by ultrafiltration using concentrator columns (cat. no 89884A; Thermo Fisher Scientific, Waltham, MA, USA) and the elution buffer was successively replaced by PBS. The final protein concentration was determined using a Bradford assay (cat. no. 500-0006; Bio-Rad Laboratories, Inc., Hercules, CA, USA) and Coomassie blue gel staining.

### Fluorescence quenching

To assess the binding of DARPin E2_79 to IgE, human recombinant FcεRI (cat. no. F0025-07; US Biological, Salem, MA, USA) was immobilized at 2 µg mL^−1^ on an ELISA plate. For the binding specificity, DARPin E2_79 was pre-incubated for 5 min with 1 µg mL^−1^ IgE with increasing concentrations of DARPin E2_79 (0-fold, 5-fold, 10-fold, 20-fold, and 100-fold molar excess over IgE). For the disruption of the IgE-FcεRI complex, IgE was added to each well at a 1 µg mL^−1^ concentration, incubated for 30 min at room temperature and subsequently washed. Increasing concentrations of DARPin E2_79 were added (5, 20, and 50 mM, corresponding to a 750, 3000, 7500-fold molar excess over IgE, respectively), incubated for 2 h and removed by washing. Then, an anti-human Ig kappa light-chain FITC (cat. no. 11-9970-42; Affymetrix eBioscience, San Diego, CA, USA; dilution 1 : 200) was added for IgE detection.

### Encapsulation and cultivation of HEK-293 cells

The encapsulation of HEK-293 cells was performed as previously described^35^. Briefly, coherent alginate-poly-(l-lysine)-alginate beads (400 µm diameter) were formed using an Inotech Encapsulator Research Unit IE 50R (Buechi Labortechnik AG, Flawil, Switzerland) set to the following parameters: 0.2 mM nozzle with a vibration frequency of 1025 Hz, 25-mL syringe operated at a flow rate of 410 units and 1.12 kV for the bead dispersion. 200 µL of microencapsulated cells (8 × 10^5^ cells, 200 cells per capsule, 4000 capsules) and 300 µL of RPMI-1640 Medium (cat. no. R8758; Sigma-Aldrich, Munich, Germany) were added to 500 µL of whole blood. Controls for constitutive production of DARPin E2_79 were run with a mock plasmid (pColaDuet-1). The plates were incubated at 37 °C in a humidified atmosphere containing 5% CO_2_. The plates were prepared for each time point (0, 24, 48 and 72 h). For the plasma collection, the plates were centrifuged for 10 min at 700 × *g* at 4 °C. The plasma samples were stored at −80 °C.

### Cell-based allergy test

The cell-based allergy test was performed as previously described^45^. In brief, 5 × 10^4^ HSD_SEAP32_ cells were seeded into each well of a 96-well plate containing 105 µL of standard cultivation medium. After 12 h, 15 µL of human serum derived from histamine release assays was added, and the cells were incubated for an additional 24 h. The supernatant was collected and either directly used for analytical assays or stored at −20 °C until further SEAP quantification.

### Basophil activation test

The basophil activation test was performed with the Flow CAST Basophil Activation Test Kit (Bühlmann Laboratories AG, Schönenbuch, Switzerland). Blood samples of allergic donors were analyzed after 24 h according to the manufacturer’s instructions.

### Quantification of the IgE–FcεRI complex dissociation

The quantification of the IgE–FcεRI complex dissociation is based on the Flow CAST Basophil Activation Test Kit. Whole-blood samples were incubated overnight with increasing concentrations of DARPin E2_79 (0, 5, 10, and 20 µM). A staining reagent containing anti-Human CD193 (CCR3) PE (cat. no. 12-1939-42, Thermo Fisher Scientific, Waltham, MA, USA) and anti-Human Ig kappa light-chain FITC (cat. no. 11-9970-42; Affymetrix eBioscience) was added to the blood samples according to the manufacturer’s instructions instead of the Flow CAST Basophil Activation Test Kit Staining Reagent. Samples were measured after 24 h according to the manufacturer’s instructions.

### Allergens

Crude allergen extracts, including including Betula alba pollen (white birch, cat. no. P6770, Sigma), Corylis avellana pollen (hazel, cat. no. 0127, Allergon AB, Ängelholm, Sweden), Cynodon dactylon pollen (Bermuda grass, cat. no. 0421, Allergon AB), Phleum pratense (timothy grass, cat. no. 0113, Allergon AB), Lolium perenne pollen (perennial rye grass, cat. no. 0214, Allergon AB), Dermatophagoides pteronyssinus allergen (house dust mite, cat. no. 4965, Allergon AB), were dissolved in degranulation buffer (25mM piperazine-N,N0-bis(2-ethanesulfonic acid) (cat. no. 80635, Sigma), 110mM sodium chloride (cat. no. S7653, Sigma), 5mM potassium chloride (cat. no. P9541, Sigma) and 0.01% (v/v) thimerosal (cat. no. T8784, Sigma); pH 7.4), and each mixture was stirred at 300 r.p.m. at 4 °C for 16 h. Subsequently, each allergen mixture was centrifuged, the supernatant was filter-sterilized and the total protein concentration was measured using the Bradford assay. The allergen solutions were stored at −20 °C at a concentration of 1 mg mL^−1^ ^45^.

### Human samples

Peripheral blood was drawn from allergic and non-allergic donors after obtaining their informed consent. The study was approved by the Ethics Committee of ETH Zürich (no. EK 2012-N-42).

### Data availability

All data and materials are available upon request.

## Electronic supplementary material

Supplementary Information

## References

[1] Dold S, Wjst M, von Mutius E, Reitmeir P, Stiepel E (1992). Genetic risk for asthma, allergic rhinitis, and atopic dermatitis. Arch. Dis. Child.

[2] McGowan EC (2015). Influence of early-life exposures on food sensitization and food allergy in an inner-city birth cohort. J. Allergy Clin. Immunol..

[3] Ben-Shoshan M (2010). A population-based study on peanut, tree nut, fish, shellfish, and sesame allergy prevalence in Canada. J. Allergy Clin. Immunol..

[4] Waserman S, Watson W (2011). Food allergy. Allergy Asthma Clin. Immunol..

[5] DeKruyff RH, Fang Y, Umetsu DT (1992). IL-4 synthesis by in vivo primed keyhole limpet hemocyanin-specific CD4+ T cells. I. Influence of antigen concentration and antigen-presenting cell type. J. Immunol..

[6] Germain RN (1994). MHC-dependent antigen processing and peptide presentation: Providing ligands for T lymphocyte activation. Cell.

[7] Galli SJ, Tsai M, Piliponsky AM (2008). The development of allergic inflammation. Nature.

[8] Tepper RI (1990). IL-4 induces allergic-like inflammatory disease and alters T cell development in transgenic mice. Cell.

[9] Shimoda K (1996). Lack of IL-4-induced Th2 response and IgE class switching in mice with disrupted State6 gene. Nature.

[10] Kraft S, Kinet J-P (2007). New developments in Fc[epsi]RI regulation, function and inhibition. Nat. Rev. Immunol..

[11] Turner H, Kinet J-P (1999). Signalling through the high-affinity IgE receptor Fc[epsi]RI. Nature.

[12] Daëron M, Malbec O, Latour S, Arock M, Fridman WH (1995). Regulation of high-affinity IgE receptor-mediated mast cell activation by murine low-affinity IgG receptors. J. Clin. Invest..

[13] Howarth PH (2000). Allergic rhinitis: not purely a histamine-related disease. Allergy.

[14] Taube C (2002). The role of IL-13 in established allergic airway disease. J. Immunol..

[15] Mattes J (2001). IL-13 induces airways hyperreactivity independently of the IL-4R alpha chain in the allergic lung. J. Immunol..

[16] Kumar RK (2002). Role of interleukin-13 in eosinophil accumulation and airway remodelling in a mouse model of chronic asthma. Clin. Exp. Allergy..

[17] Li L (1999). Effects of Th2 cytokines on chemokine expression in the lung: IL-13 potently induces eotaxin expression by airway epithelial cells. J. Immunol.

[18] Walter DM (2001). Critical role for IL-13 in the development of allergen-induced airway hyperreactivity. J. Immunol..

[19] Kawakami T, Galli SJ (2002). Regulation of mast-cell and basophil function and survival by IgE. Nat. Rev. Immunol..

[20] Simon FE, Simons K (2008). H1 antihistamines: Current status and future directions. World Allergy Organization J..

[21] White MV (1990). The role of histamine in allergic diseases. J. Allergy Clin. Immunol..

[22] Thurmond RL, Gelfand EW, Dunford PJ (2008). The role of histamine H1 and H4 receptors in allergic inflammation: the search for new antihistamines. Nat. Rev. Drug. Discov..

[23] Jutel M, Akdis CA (2011). Immunological mechanisms of allergen-specific immunotherapy. Allergy.

[24] Winther L, Reimert C, Skov P, Kærgaard Poulsen L, Moseholm L (1999). Basophil histamine release, IgE, eosinophil counts, ECP, and EPX are related to the severity of symptoms in seasonal allergic rhinitis. Allergy.

[25] Kobayashi Y (1994). Predictive values of cord blood IgE and cord blood lymphocyte responses to food antigens in allergic disorders during infancy. J. Allergy Clin. Immunol..

[26] Hans D (2014). Quilizumab is an afucosylated humanized anti-M1 prime therapeutic antibody. Clin. Antiinflamm. Antiallergy Drugs.

[27] Harris JM (2016). A randomized trial of the efficacy and safety of quilizumab in adults with inadequately controlled allergic asthma. Respir. Res..

[28] Molimard M, de Blay F, Didier A, Le Gros V (2008). Effectiveness of omalizumab (Xolair®) in the first patients treated in real-life practice in France. Respir. Med..

[29] Oba Y, Salzman GA (2004). Cost-effectiveness analysis of omalizumab in adults and adolescents with moderate-to-severe allergic asthma. J. Allergy Clin. Immunol..

[30] Binz HK, Stumpp MT, Forrer P, Amstutz P, Plückthun A (2003). Designing repeat proteins: Well-expressed, soluble and stable proteins from combinatorial libraries of consensus ankyrin repeat proteins. J. Mol. Biol..

[31] Binz HK (2004). High-affinity binders selected from designed ankyrin repeat protein libraries. Nat. Biotech..

[32] Kim B (2012). Accelerated disassembly of IgE-receptor complexes by a disruptive macromolecular inhibitor. Nature.

[33] Baumann MJ, Eggel A, Amstutz P, Stadler BM, Vogel M (2010). DARPins against a functional IgE epitope. Immunol. Lett..

[34] Rössger K, Charpin-El Hamri G, Fussenegger M (2013). Reward-based hypertension control by a synthetic brain–dopamine interface. Proc. Natl Acad. Sci. USA.

[35] Ye H, Baba MD-E, Peng R-W, Fussenegger M (2011). A synthetic optogenetic transcription device enhances blood-glucose homeostasis in mice. Science.

[36] Shirakawa T (2011). Deactivation of STAT6 through serine 707 phosphorylation by JNK. J. Biol. Chem..

[37] Hebenstreit D, Luft P, Schmiedlechner A, Duschl A, Horejs-Hoeck J (2005). SOCS-1 and SOCS-3 inhibit IL-4 and IL-13 induced activation of Eotaxin-3/CCL26 gene expression in HEK293 cells. Mol. Immunol..

[38] Murata T, Taguchi J, Puri RK (1998). Interleukin-13 receptor α′ but not α chain: A functional component of interleukin-4 receptors. Blood.

[39] Wang HY, Zamorano J, Yoerkie JL, Paul WE, Keegan AD (1997). The IL-4-induced tyrosine phosphorylation of the insulin receptor substrate is dependent on JAK1 expression in human fibrosarcoma cells. J. Immunol..

[40] Leonard C, Tormey V, Burke C, Poulter LW (1997). Allergen-induced cytokine production in atopic disease and its relationship to disease severity. Am. J. Respir. Cell Mol. Biol..

[41] Wong CK (2001). Proinflammatory cytokines (IL-17, IL-6, IL-18 and IL-12) and Th cytokines (IFN-γ, IL-4, IL-10 and IL-13) in patients with allergic asthma. Clin. Exp. Immunol..

[42] Mueller TD, Zhang J-L, Sebald W, Duschl A (2002). Structure, binding, and antagonists in the IL-4/IL-13 receptor system. Biochim. Biophys. Acta Mol. Cell Res..

[43] LaPorte SL (2008). Molecular and structural basis of cytokine receptor pleiotropy in the interleukin-4/13 system. Cell.

[44] Eggel A (2011). Inhibition of ongoing allergic reactions using a novel anti-IgE DARPin-Fc fusion protein. Allergy.

[45] Ausländer D (2014). A designer cell-based histamine-specific human allergy profiler. Nat. Commun..

[46] MacGlashan DW (2013). Basophil activation testing. J. Allergy Clin. Immunol..

[47] Eggel A (2014). Accelerated dissociation of IgE-FcεRI complexes by disruptive inhibitors actively desensitizes allergic effector cells. J. Allergy Clin.Immunol..

[48] Magnan A (2000). Relationships between natural T cells, atopy, IgE levels, and IL-4 production. Allergy.

[49] Weber W, Fussenegger M (2012). Emerging biomedical applications of synthetic biology. Nat. Rev. Genet..

[50] Ausländer S, Fussenegger M (2013). From gene switches to mammalian designer cells: present and future prospects. Trends Biotechnol..

[51] Auslander S, Auslander D, Muller M, Wieland M, Fussenegger M (2012). Programmable single-cell mammalian biocomputers. Nature.

[52] Ausländer D (2014). A synthetic multifunctional Mammalian pH sensor and CO2 transgene-control device. Mol. Cell.

[53] Muller M, Auslander S, Auslander D, Kemmer C, Fussenegger M (2012). A novel reporter system for bacterial and mammalian cells based on the non-ribosomal peptide indigoidine. Metab. Eng..

[54] Muller M (2017). Designed cell consortia as fragrance-programmable analog-to-digital converters. Nat. Chem. Biol..

[55] Ye H (2013). Pharmaceutically controlled designer circuit for the treatment of the metabolic syndrome. Proc. Natl Acad. Sci. USA.

[56] Siergiejko Z (2011). Oral corticosteroid sparing with omalizumab in severe allergic (IgE-mediated) asthma patients. Curr. Med. Res. Opin..

[57] McFadden ER (2003). Acute severe asthma. Am. J. Respir. Crit. Care. Med..

[58] Bayes HK, Thomson NC (2016). Acute severe asthma in adults. Medicine.

[59] Ng KH (2004). Central nervous system side effects of first- and second-generation antihistamines in school children with perennial allergic rhinitis: A randomized, double-blind, placebo-controlled comparative study. Pediatrics.

[60] Van Ganse E (1997). Effects of antihistamines in adult asthma: a meta-analysis of clinical trials. Eur. Respir. J..

[61] Djukanovic R, Hanania N, Busse W, Price D (2016). IgE-mediated asthma: New revelations and future insights. Respir. Med..

[62] Fischbach MA, Bluestone JA, Lim WA (2013). Cell-based therapeutics: The next pillar of medicine. Sci. Transl. Med..

[63] Saxena P (2016). A programmable synthetic lineage-control network that differentiates human IPSCs into glucose-sensitive insulin-secreting beta-like cells. Nat. Commun..

[64] Xie M (2016). *β*-cell–mimetic designer cells provide closed-loop glycemic control. Science.

[65] Taylor A, Verhagen J, Blaser K, Akdis M, Akdis CA (2006). Mechanisms of immune suppression by interleukin-10 and transforming growth factor-*β*: the role of T regulatory cells. Immunology.

[66] Finkelman FD (1988). IL-4 is required to generate and sustain in vivo IgE responses. J. Immunol..

[67] Ying S, Meng Q, Corrigan CJ, Lee TH (2006). Lack of filaggrin expression in the human bronchial mucosa. J. Allergy Clin. Immunol..

[68] Bitoun E (2002). Netherton syndrome: Disease expression and spectrum of SPINK5 mutations in 21 families. J. Invest. Dermatol..

[69] Wills-Karp M, Ewart SL (2004). Time to draw breath: asthma-susceptibility genes are identified. Nat. Rev. Genet..

[70] Dombrowicz D (1996). Anaphylaxis mediated through a humanized high affinity IgE receptor. J. Immunol..

[71] Yamaguchi M (1997). IgE enhances mouse mast cell FcεRI expression in vitro and in vivo: Evidence for a novel amplification mechanism in IgE-dependent reactions. J. Exp. Med..

[72] Foster B, Metcalfe DD, Prussin C (2003). Human dendritic cell 1 and dendritic cell 2 subsets express FcεRI: Correlation with serum IgE and allergic asthma. J. Allergy Clin. Immunol..

[73] Greer AM (2014). Serum IgE clearance is facilitated by human FcεRI internalization. J. Clin. Invest..

[74] Holgate ST, Djukanovic R, Casale T, Bousquet J (2005). Anti-immunoglobulin E treatment with omalizumab in allergic diseases: an update on anti-inflammatory activity and clinical efficacy. Clin. Exp. Allergy.

[75] Lin H (2004). Omalizumab rapidly decreases nasal allergic response and FcepsilonRI on basophils. J. Allergy Clin. Immunol..

[76] Skoner DP (2001). Allergic rhinitis: Definition, epidemiology, pathophysiology, detection, and diagnosis. J. Allergy Clin. Immunol..

[77] Stahl A (2013). Highly potent VEGF-A-antagonistic DARPins as anti-angiogenic agents for topical and intravitreal applications. Angiogenesis.

[78] Stumpp MT, Binz HK, Amstutz P (2008). DARPins: A new generation of protein therapeutics. Drug Discov. Today.

[79] Simonsen JL (2002). Telomerase expression extends the proliferative life-span and maintains the osteogenic potential of human bone marrow stromal cells. Nat. Biotechnol..

[80] Schlatter S, Rimann M, Kelm J, Fussenegger M (2002). SAMY, a novel mammalian reporter gene derived from Bacillus stearothermophilus α-amylase. Gene.

