# Supplementary material for: Sensing and responding to allergic response cytokines through a genetically encoded circuit
Source: Nat Commun. 2017 Oct 24;8:1101. doi: 10.1038/s41467-017-01211-1 (PMC5653676; doi:10.1038/s41467-017-01211-1)
Supplement: Supplementary file 1 — Supplementary Information [file 41467_2017_1211_MOESM1_ESM.pdf]

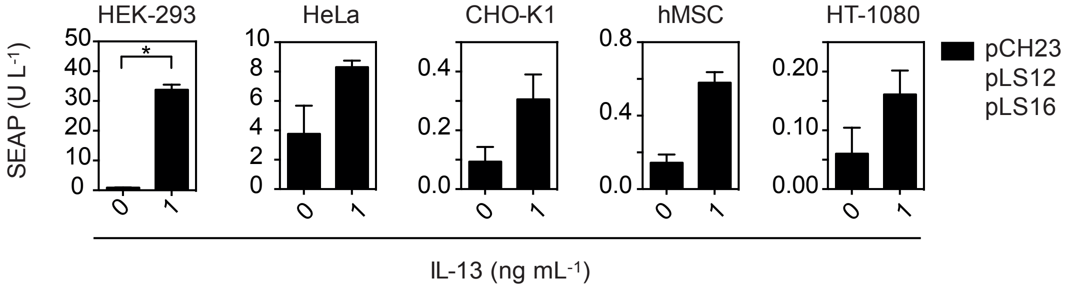


**Supplementary Figure 1 | Performance of the DCS device in different mammalian cell lines.**

HEK-293, HeLa, CHO-K1, hMSC, and HT-1080 were co-transfected with expression vectors encoding the IL-13Rα1 receptor subunit (pCH23, $P_{hCMV}$-*IL13RA1*-pA), the STAT6 transcription factor (pLS16, $P_{hCMV}$-*STAT6*-pA) and the SEAP reporter gene (pLS12, $P_{STAT6/cEBP}$-*SEAP*-pA), and SEAP was profiled in the culture supernatant after 48 h. The data represent the means ± s.d. (n≥3 experiments). *$P < 0.05$, two-tailed Student's t test.

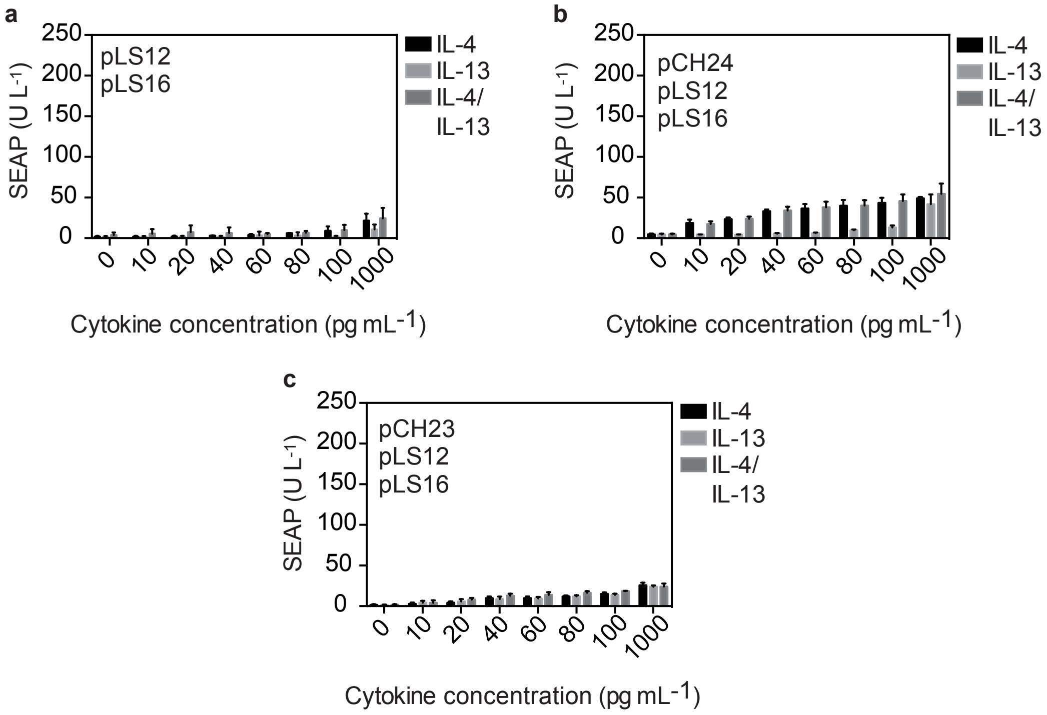


**d**

| | IL-13 | | IL-4 | | IL-4 and IL-13 | |
|---|---|---|---|---|---|---|
| **Figure** | **Estimated slope** | **P value** | **Estimated slope** | **P value** | **Estimated slope** | **P value** |
| 1c | 0.0381 | 0.13 | 0.0128 | 0.55 | 0.0253 | 0.32 |
| 1d | 27.3 | 7.5e-19 | 26.4 | 2.1e-23 | 30.5 | 4.3e-38 |
| 1e | -0.0117 | 0.74 | 0.0547 | 0.00043 | 0.0451 | 0.17 |
| 1f | 0.66 | 0.0092 | 0.374 | 0.079 | 0.498 | 0.017 |
| Supplementary Fig. 2a | 1.45 | 0.00015 | 3.42 | 1.7e-11 | 3.22 | 6e-06 |
| Supplementary Fig. 2b | 8.46 | 8.7e-16 | 6.85 | 1.8e-15 | 8.23 | 2e-13 |
| Supplementary Fig. 2c | 4.43 | 2e-28 | 5.17 | 6.7e-34 | 4.49 | 1.1e-17 |

**Supplementary Figure 2 | Design and validation of the dual $T_H2$ cytokine sensor (DCS) device.**

(**a**) DCS performance in the absence of heterologous expression of IL-13 receptor components. 3 x $10^4$ HEK-293 cells were transfected with the STAT6 transcription factor

(pLS16, $P_{hCMV}$-*STAT6*-pA) and the SEAP expression vector pLS12 ($P_{STAT6/cEBP}$-*SEAP*-pA) and exposed for 48 h to different physiological cytokine concentrations (IL-4, IL-13, or both) before SEAP levels were profiled in the culture supernatant. (**b**) DCS performance in the absence of heterologous expression of the IL-13Rα1 receptor subunit. 3 x $10^4$ HEK-293 co-transfected with pCH24 ($P_{hCMV}$-*IL4RA*-pA), pLS16 ($P_{hCMV}$-*STAT6*-pA), and pLS12 ($P_{STAT6/cEBP}$-*SEAP*-pA) were cultivated for 48 h in the presence of different physiological cytokine concentrations (IL-4, IL-13, or both) before SEAP was quantified in the culture supernatant. (**c**) DCS performance in the absence of heterologous expression of the IL-4Rα receptor subunit. 3 x $10^4$ HEK-293 co-transfected with pCH23 ($P_{hCMV}$-*IL13RA1*-pA), pLS16 ($P_{hCMV}$-*STAT6*-pA), and pLS12 ($P_{STAT6/cEBP}$-*SEAP*-pA) were cultivated for 48 h in the presence of different physiological cytokine concentrations (IL-4, IL-13, or both) before SEAP was quantified in the culture supernatant. The data represent the means ± s.d. (n≥3 experiments). (**d**) A mathematical model was developed for the analysis of the in vitro results of Fig.1 and Supplementary Figure 1 and 2, by estimating the statistical significance of the correlation between SEAP and the log of the concentration ($\beta_1$), following the equation: SEAP = $\beta_1 \times$ log(concentration) + $\beta_2 \times$ experiment + error. The model is based on the data of three independent experiments (including at least two technical replicates per experiment) performed for each figure.

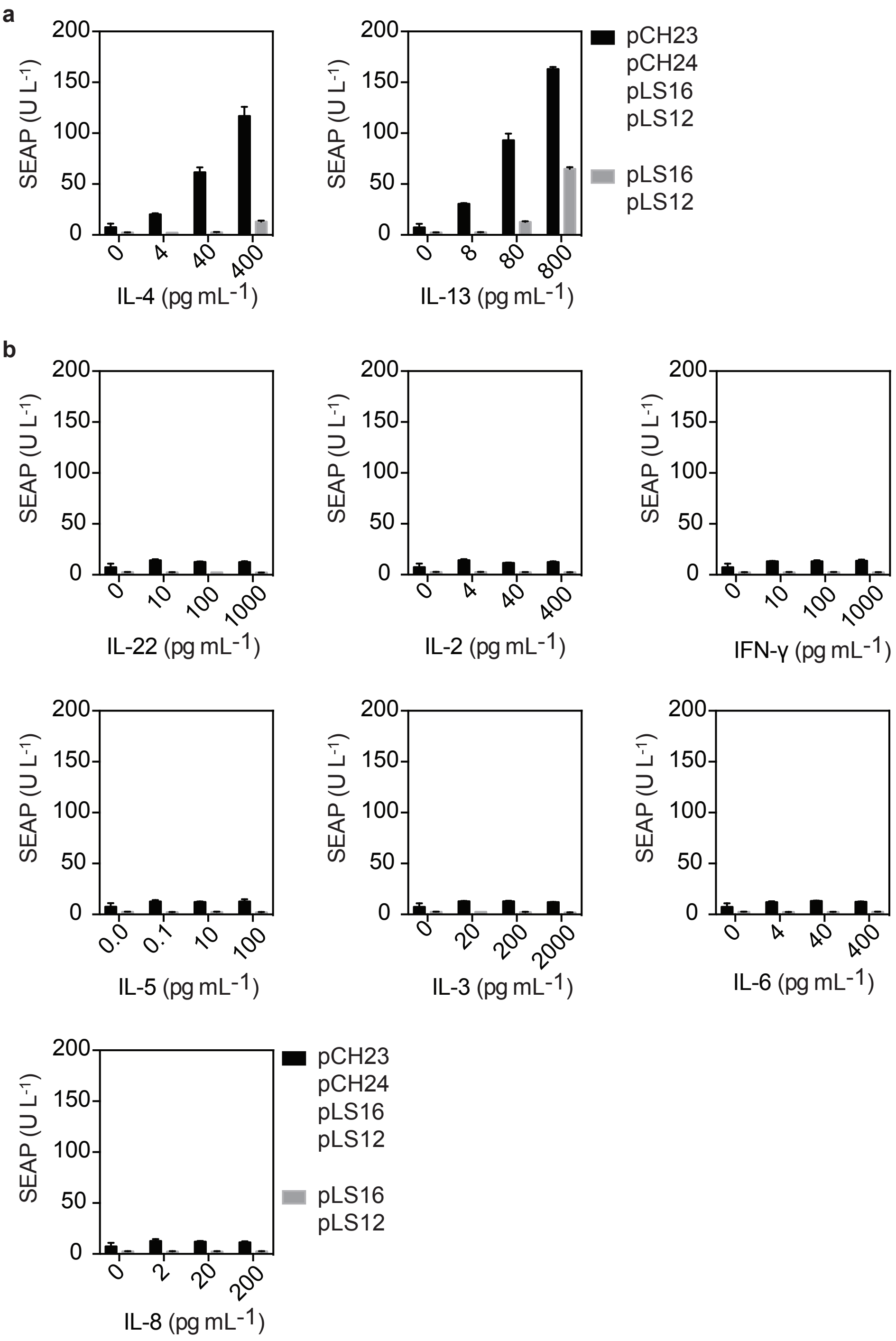


**Supplementary Figure 3 | The IL-13R is specifically responsive to the cytokines IL-4 and IL-13.**

DCS cells (black bars) co-transfected with the expression vectors encoding the IL-13R receptor subunits pCH23 ($P_{hCMV}$-*IL13RA1*-pA) and pCH24 ($P_{hCMV}$-*IL4RA*-pA), the STAT6 transcription factor (pLS16, $P_{hCMV}$-*STAT6*-pA) and the SEAP reporter gene (pLS12, $P_{STAT6/cEBP}$-*SEAP*-pA), and control cells (grey bars) co-transfected with the expression vectors encoding the STAT6 transcription factor (pLS16, $P_{hCMV}$-*STAT6*-pA) and the SEAP reporter

gene (pLS12, $P_{STAT6/cEBP}$-*SEAP*-pA), exposed (**a**) to increasing concentrations of IL-4 and IL-13, and (**b**) to increasing concentrations of IL-22, IL-2, IFN-γ, IL-5, IL-3, IL-6, and IL-8, before SEAP levels were profiled in the culture supernatant after 48 h.

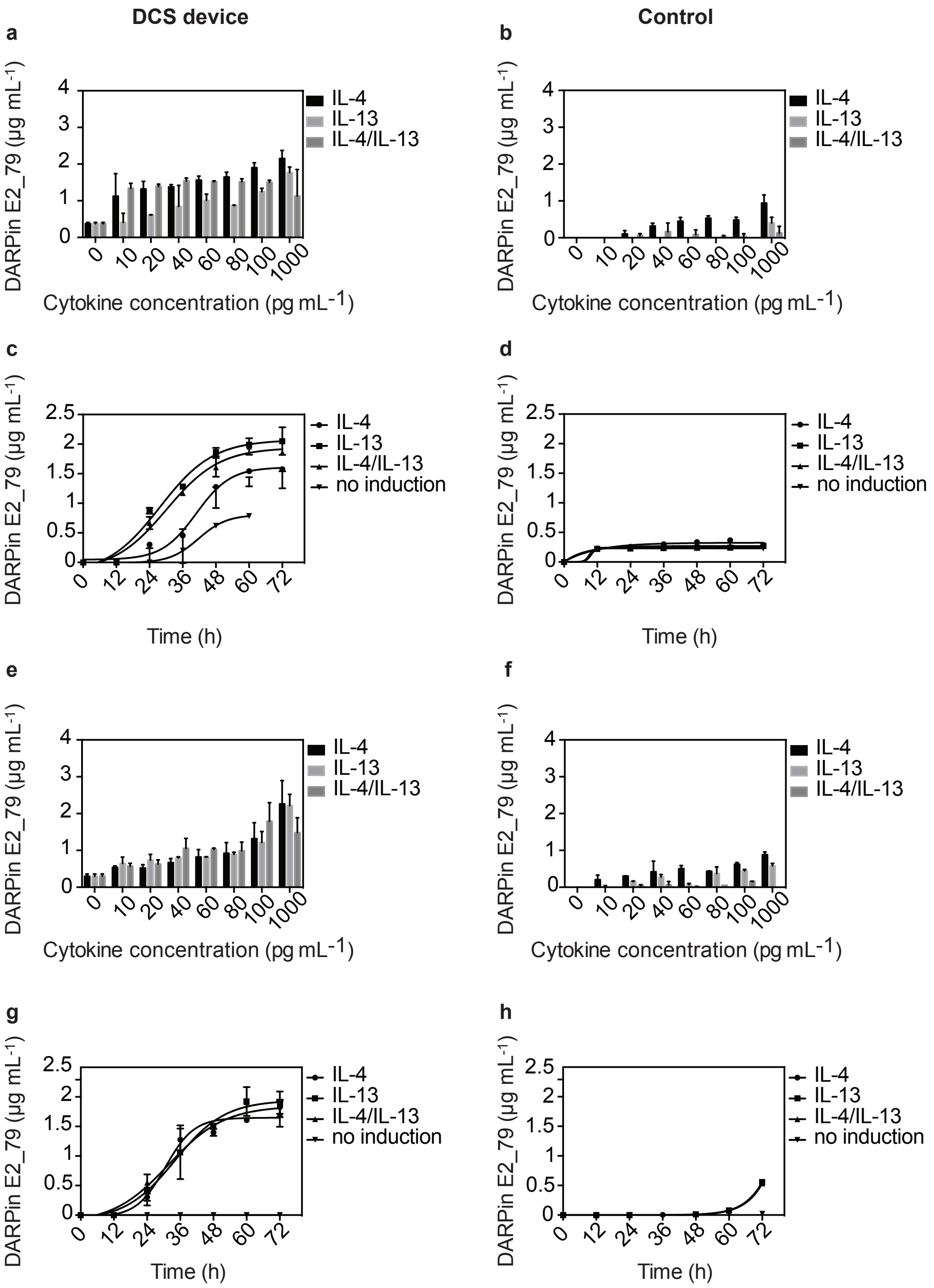


**Supplementary Figure 4 | *In vitro* and *ex vivo* dose-dependent DARPin E2_79 expression and DARPin E2_79 expression kinetics profiles of the DCS device.**

*In vitro* dose-dependent DARPin E2_79 expression profiles. (**a**) DCS-controlled DARPin E2_79 (200 cells per capsule, 4000 capsules) and (**b**) 8 x $10^5$ microencapsulated HEK-293 control cells lacking the DCS IL13R consisting of pCH23 ($P_{hCMV}$-*IL13RA1*-pA) and pCH24 ($P_{hCMV}$-*IL4RA*-pA), were cultivated for 48 h in the presence of different physiological

cytokine concentrations (IL-4, IL-13, or both) before DARPin E2_79 was quantified in the culture supernatant. *In vitro* DARPin E2_79 kinetic expression profiles: (**c**) 8 x $10^5$ microencapsulated HEK-293 cells transgenic for DCS-controlled DARPin E2_79 (200 cells per capsule, 4000 capsules) and (**d**) 8 x $10^5$ microencapsulated HEK-293 control cells lacking the DCS IL13R consisting of pCH23 ($P_{hCMV}$-*IL13RA1*-pA) and pCH24 ($P_{hCMV}$-*IL4RA*-pA), were cultivated for 72 h in the presence of pathophysiological cytokine concentrations (IL-4, 40 pg $mL^{-1}$ and IL-13, 80 pg $mL^{-1}$) while DARPin E2_79 levels were profiled in the culture supernatant at 12 h intervals. *Ex vivo* dose-dependent DARPin E2_79 expression profiles: (**e**) 8 x $10^5$ microencapsulated HEK-293 cells transgenic for DCS-controlled DARPin E2_79 (200 cells per capsule, 4000 capsules) and (**f**) 8 x $10^5$ microencapsulated HEK-293 control cells lacking the DCS IL13R consisting of pCH23 ($P_{hCMV}$-*IL13RA1*-pA) and pCH24 ($P_{hCMV}$-*IL4RA*-pA), were cultivated for 48h in in non-allergic human whole blood diluted 1:1 in RPMI-1640 the presence of different physiological cytokine concentrations (IL-4, IL-13, or both) before DARPin E2_79 was quantified in the culture supernatant. *Ex vivo* DARPin E2_79 kinetic expression profiles: (**g**) 8 x $10^5$ microencapsulated HEK-293 cells transgenic for DCS-controlled DARPin E2_79 (200 cells per capsule, 4000 capsules) and (**h**) 8 x $10^5$ microencapsulated HEK-293 control cells lacking the DCS IL13R consisting of pCH23 ($P_{hCMV}$-*IL13RA1*-pA) and pCH24 ($P_{hCMV}$-*IL4RA*-pA), were cultivated for 48h in non-allergic human whole blood diluted 1:1 in RPMI-1640 for 72 h in the presence of pathophysiological cytokine concentrations (IL-4, 40 pg $mL^{-1}$ and IL-13, 80 pg $mL^{-1}$) while DARPin E2_79 levels were profiled in the culture supernatant at 12 h intervals.

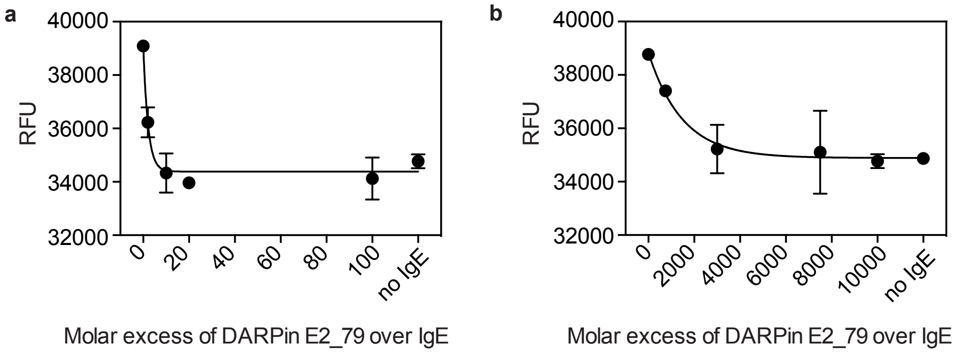


**Supplementary Figure 5 | Binding and dissociation properties of DARPin E2_79 produced in mammalian cells.**

(**a**) Binding of DARPin E2_79 to IgE prevents the binding of IgE to the immobilized FcεRI, therefore quenching the fluorescent signal (RFU, relative fluorescence units) with increasing concentrations. (**b**) Increasing DARPin E2_79 concentrations dissociate the IgE from the immobilized FcεRI receptor and quench the fluorescent signal (RFU, relative fluorescence units) of IgE bound to the FcεRI.

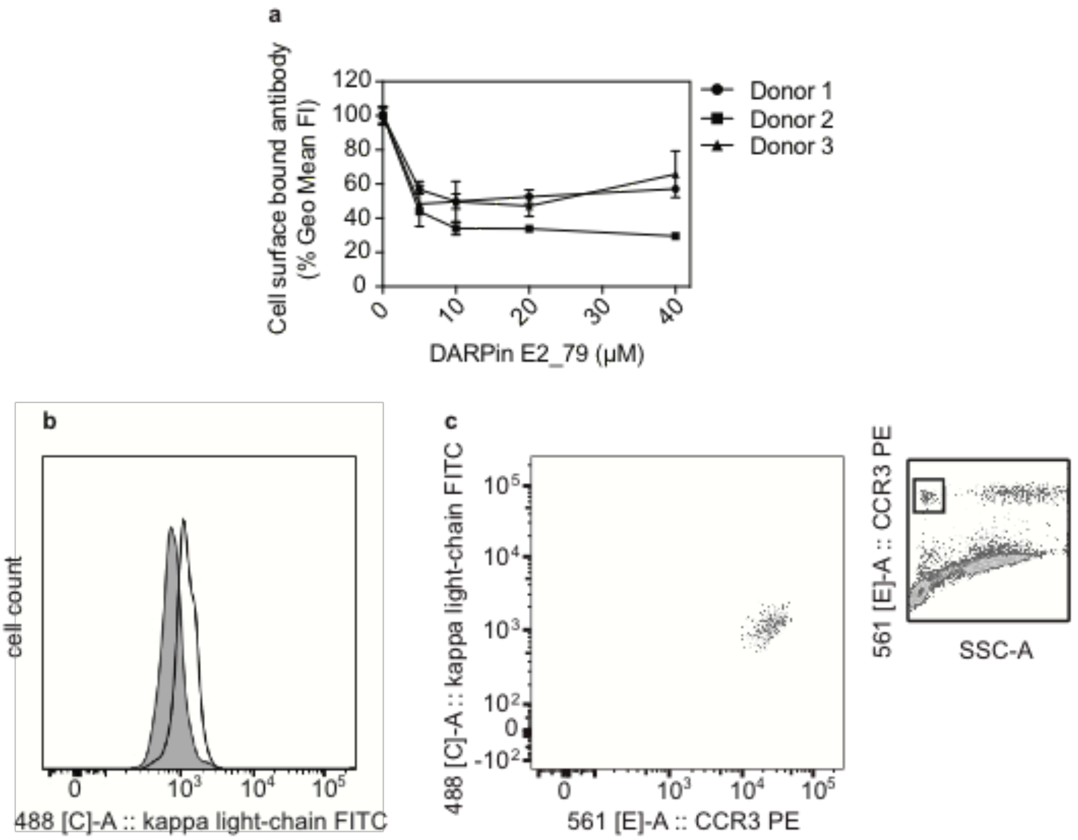


**Supplementary Figure 6 | Dose-dependent desensitization of whole blood basophil granulocytes by DARPin E2_79 produced in mammalian cells.**

(**a**) The addition of increasing concentrations of mammalian produced DARPin E2_79 reduces the amount of IgE on the surface of whole blood basophils. Surface antibody levels were quantified by flow cytometric analysis after 24h. Data are means ± s.d. of each blood donor sample (n = 3) measured in duplicate. (**b**) The histogram shows the removal of IgE from the surface of blood basophils for one donor at the concentrations of 0 µM (white histogram) and 40 µM (shaded histogram) DARPin E2_79. (**c**) Flow cytometric analysis gating approach for the blood basophils, shown here for one donor at 0 µM DARPin E2_79.

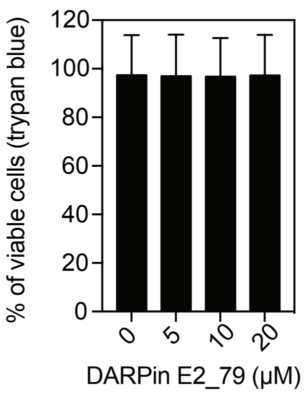


**Supplementary Figure 7 | Trypan blue-based viability assay of HEK-293 cells exposed to increasing concentrations of DARPin E2_79.**

3 x $10^4$ HEK-293 cells were transfected with expression vectors encoding the IL-13R receptor subunits (pCH23, $P_{hCMV}$-*IL13RA1*-pA, and pCH24, $P_{hCMV}$-*IL4RA*-pA), the STAT6 transcription factor (pLS16, $P_{hCMV}$-*STAT6*-pA) and the SEAP expression vector pLS12 ($P_{STAT6/cEBP}$-*SEAP*-pA).

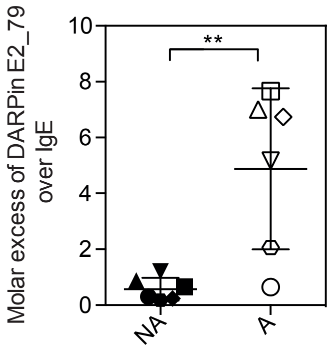


**Supplementary Figure 8 | Molar excess of DARPin E2_79 to IgE.**

Molar excess of DARPin E2_79 over IgE in the blood of non-allergic (NA) and allergic (A) donors. $^{**}P < 0.005$, two-tailed Student's t test.

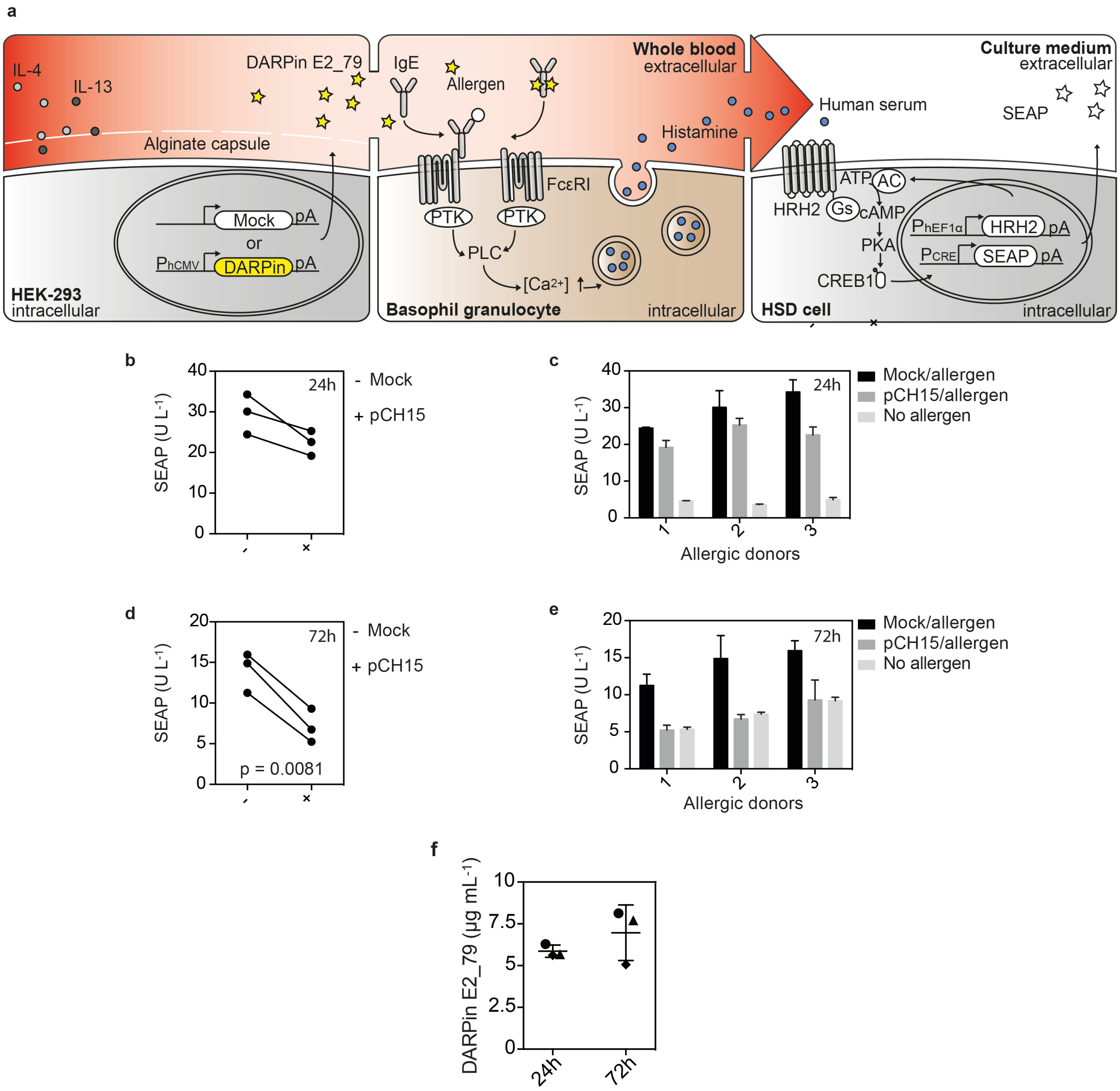


**Supplementary Figure 9 | Impact of constitutive DARPin E2_79 production on the histamine release and functionality of the blood basophil granulocytes.**

(**a**) Illustration of the operational procedure of the constitutive DARPin E2_79 production in human whole blood. (**b**) 8 x $10^5$ microencapsulated HEK-293 cells transfected with the $P_{hCMV}$-driven DARPin E2_79 expression vector pCH15 ($P_{hCMV}$-*$SS_{SEAP}$-His-DARPin E2_79*-pA) for constitutive DARPin E2_79 production (200 cells per capsule, 4000 capsules) were cultivated in 0.5 mL of human blood diluted 1:1 in RPMI-1640 for 24 h. The histamine release of the blood basophil granulocytes was reduced when the DARPin E2_79 was produced constitutively, while it remained high when the cells were transfected with a mock plasmid. Data are means ± s.d. of each blood donor sample (n = 3) measured in duplicate. (**c**) Allergen-induced histamine release (mock plasmid or pCH15) compared to basal levels of

histamine (no addition of allergen) show that the basophil granulocytes in the whole blood cell culture are functional after 24h. (**d**) After 72h, the histamine release is significantly reduced for the same blood donors shown in (**a**). Paired t-test: $t(2) = 11.02$, $p = 0.0081$. (**e**) Allergen-induced histamine release (mock plasmid or pCH15) compared to basal levels of histamine (no addition of allergen) show that the basophil granulocytes in the whole blood cell culture are functional after 72h. (**f**) DARPin E2_79 production is shown for the three blood donors after 24h and 72h.

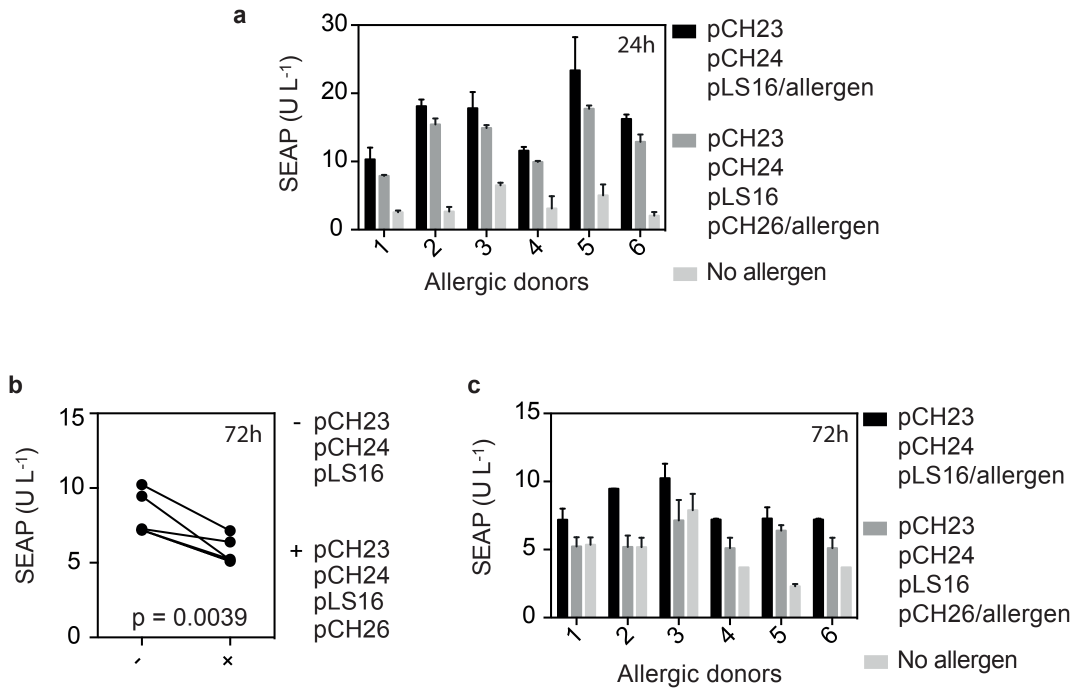


**Supplementary Figure 10 | Reduction of the histamine release and functionality of the basophil granulocytes.**

(**a**) Allergen-induced histamine release of microencapsulated HEK-293 cells transgenic for DCS-controlled DARPin E2_79 production and control cells lacking DCS DARPin E2_79 reporter gene pCH26 ($P_{STAT6/cEBP}$-*SS<sub></sub>*$_{SEAP}$-*His-DARPin E2_79*-pA) (see Fig. 4a, V) compared to basal levels of histamine (no addition of allergen) show that the basophils granulocytes in the whole blood cell culture are functional after 24h. (**b**) The reduction of the histamine release upon allergen trigger is significantly reduced after 72h for the same blood donors than shown in Fig. 4a, V and in (**a**). Paired t-test: $t(5) = 5.066$, $p = 0.0039$. (**c**) Allergen-induced histamine release (HEK-293 cells transgenic for DCS-controlled DARPin E2_79 production and control cells lacking DCS DARPin E2_79 reporter gene pCH26 ($P_{STAT6/cEBP}$-*SS*$_{SEAP}$-*His-DARPin E2_79*-pA)) compared to basal levels of histamine (no addition of allergen) show that the basophil granulocytes in the whole blood cell culture are functional after 72h.

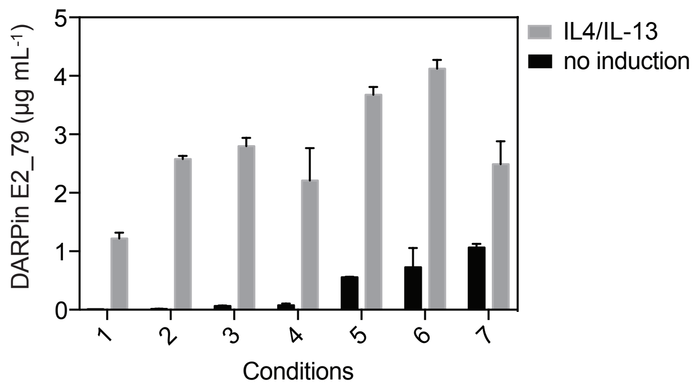


**Supplementary Figure 11 | Optimization of the DCS expression levels of DARPin E2_79.** 3 x $10^4$ HEK-293 cells were co-transfected with the expression vectors encoding the IL-13Rα1 receptor subunit (pCH23, $P_{hCMV}$-*IL13RA1*-pA) and (pCH24, $P_{hCMV}$-*IL4RA*-pA), the STAT6 transcription factor (pLS16, $P_{hCMV}$-*STAT6*-pA) and the DARPin E2_79 reporter gene pCH26 ($P_{STAT6/cEBP}$-$SS_{SEAP}$-*His-DARPin E2_79*-pA), and were cultivated for 24 h in the presence of pathophysiological cytokine concentrations (IL-4, 40 pg $mL^{-1}$ and IL-13, 80 pg $mL^{-1}$) before DARPin E2_79 was quantified in the culture supernatant. Condition no. 1 is the standard condition used previously, while condition no. 3 was chosen for further experiments. The data represent the means ± s.d. of technical duplicates in the ELISA.

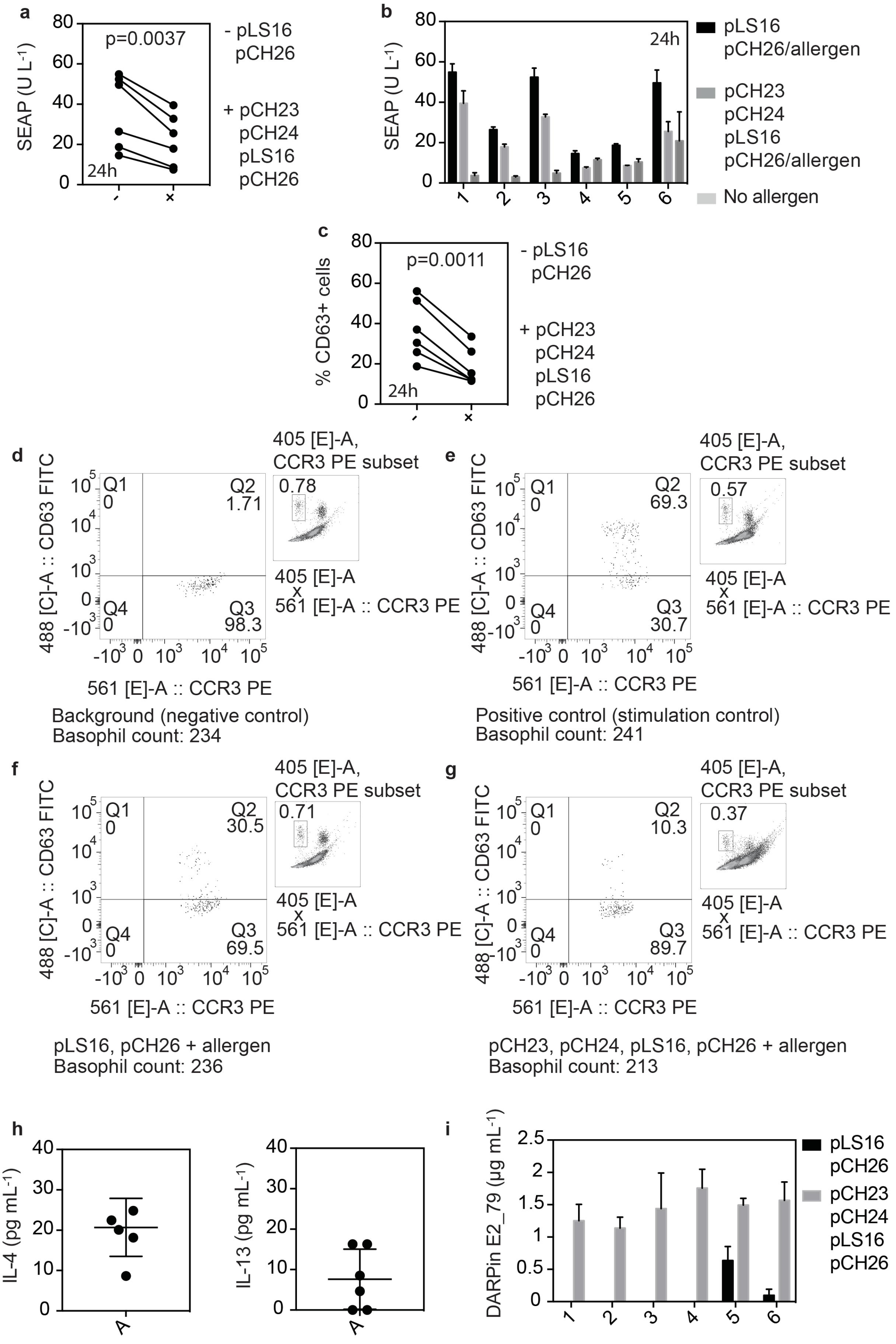
a
p=0.0037
SEAP (U L-1)
24h
- pLS16
pCH26
+ pCH23
pCH24
pLS16
pCH26
b
SEAP (U L-1)
24h
pLS16
pCH26/allergen
pCH23
pCH24
pLS16
pCH26/allergen
No allergen
c
p=0.0011
% CD63+ cells
24h
- pLS16
pCH26
+ pCH23
pCH24
pLS16
pCH26
d
405 [E]-A,
CCR3 PE subset
0.78
488 [C]-A :: CD63 FITC
Q1 0
Q2 1.71
Q4 0
Q3 98.3
405 [E]-A
x
561 [E]-A :: CCR3 PE
561 [E]-A :: CCR3 PE
Background (negative control)
Basophil count: 234
e
405 [E]-A,
CCR3 PE subset
0.57
Q1 0
Q2 69.3
Q4 0
Q3 30.7
Positive control (stimulation control)
Basophil count: 241
f
405 [E]-A,
CCR3 PE subset
0.71
Q1 0
Q2 30.5
Q4 0
Q3 69.5
pLS16, pCH26 + allergen
Basophil count: 236
g
405 [E]-A,
CCR3 PE subset
0.37
Q1 0
Q2 10.3
Q4 0
Q3 89.7
pCH23, pCH24, pLS16, pCH26 + allergen
Basophil count: 213
h
IL-4 (pg mL-1)
IL-13 (pg mL-1)
A
i
DARPin E2_79 (µg mL-1)
pLS16
pCH26
pCH23
pCH24
pLS16
pCH26

**Supplementary Figure 12 | Impact of the optimized DCS device on the reduction of the histamine release and the basophil granulocytes activation.**

(**a**) Allergen-induced histamine release of optimized (condition no 3, Supplementary Figure 11) microencapsulated HEK-293 cells transgenic for DCS-controlled DARPin E2_79 production. DCS-controlled DARPin E2_79 were cultivated in 0.5 mL of human blood diluted 1:1 in RPMI-1640 for 24 h during which the allergen-associated cytokines IL-4 and IL-13 in the donor's blood trigger DCS-controlled production and secretion of DARPin E2_79. Allergen-induced histamine release of microencapsulated HEK-293 cells transgenic for DCS-controlled DARPin E2_79 production and control cells lacking the DCS IL13R consisting of pCH23 ($P_{hCMV}$-*IL13RA1*-pA) and pCH24 ($P_{hCMV}$-*IL4RA*-pA). The reduction of the histamine release upon allergen trigger is significantly reduced after 24h for six blood donors. Paired t-test: $t(5) = 5.124$, $p = 0.0037$. Data are means ± s.d. of each blood donor sample ($n = 6$) measured in duplicate. (**b**) Allergen-induced histamine release (HEK-293 cells transgenic for DCS-controlled DARPin E2_79 production and control cells lacking DCS DARPin E2_79 reporter gene pCH26 ($P_{STAT6/cEBP}$-*$SS_{SEAP}$-His-DARPin E2_79*-pA)) compared to basal levels of histamine (no addition of allergen) show that the basophil granulocytes in the whole blood cell culture are functional after 24h. (**c**) The occurrence of the basophil activation marker CD63 was quantified by flow cytometry analysis after 24h. The reduction of the basophil activation marker CD63 upon allergen trigger is significantly reduced after 24h for six blood donors. Paired t-test: $t(5) = 6.673$, $p = 0.0011$. Data are means ± s.d. of each blood donor sample ($n = 6$) measured in duplicate. At least 200 basophil granulocytes were counted per basophil activation test. (**d**)-(**g**) Flow cytometric analysis gating approach for the blood basophil granulocytes, shown here for one donor. (**d**) Patient background. (**e**) Stimulation control. (**f**) Stimulation of human blood containing $8 \times 10^5$ microencapsulated HEK-293 control cells lacking the DCS IL13R consisting of pCH23 ($P_{hCMV}$-*IL13RA1*-pA) and pCH24 ($P_{hCMV}$-*IL4RA*-pA) and cultivated for 24 h. (**g**) Stimulation of human blood containing $8 \times 10^5$ microencapsulated HEK-293 cells transgenic for DCS-controlled DARPin E2_79 and cultivated for 24 h. (**h**) Serum concentrations of IL-4 ($n = 5$) and IL-13 ($n = 6$). (**i**) DARPin E2_79 concentrations in the blood of the six allergic donors after 24h.

**Supplementary Table 1 | Plasmids used and designed in this study**

| Plasmid | Description and Cloning Strategy | Reference or Source |
|---|---|---|
| pColaDuet™-1 | Prokaryotic expression vector ($P_{T7/lacO}$-MCS). | Merck KGgA, Darmstadt, GER |
| pcDNA3.1(+) | Mammalian expression vector ($P_{hCMV}$-MCS-pA). | Life Technologies, Zug, CH |
| pCMV-SPORT6 | Mammalian expression vector ($P_{hCMV}$-MCS-pA). | Clontech, Mountain View, USA |
| pCR®4-TOPO® | Prokaryotic expression vector ($P_{lac}$-MCS). | Clontech, Mountain View, USA |
| pSEAP2-Control | Constitutive mammalian SEAP expression vector ($P_{SV40}$-*SEAP*-pA). | Clontech, Mountain View, USA |
| pOTB7 | Prokaryotic expression vector ($P_{T7}$-MCS). | Rubin et al., 2000[1] |
| pSTAT6 | STAT6 cDNA-containing pCMV-SPORT6 (IMAGE: IRATp970B0396D). | Lennon et al., 1996[2] |
| pUC57 | pUC19-derived bacterial expression vector. | GenScript, Piscataway, USA |
| pIL4RA | pCR4®-TOPO® containing IL4RA cDNA (Clone ID:. 9020352) | GE Healthcare, Chalfont St Giles, UK |
| pIL13RA1 | pOTB7 containing IL13RA1 cDNA (IMAGE: IRAUp969F1160D). | Lennon et al., 1996[2] |
| pWW124 | SCB1-responsive SEAP expression vector ($P_{SPA}$-*SEAP*-pA). | Weber et al., 2003[3] |
| pLS12 | $P_{STAT6/cEBP}$-driven SEAP expression vector ($P_{STAT6/cEBP}$-*SEAP*-pA; $P_{STAT6/cEBP}$, $(O_{STAT6/cEBP})_4$-$P_{hCMVmin}$). A first $(O_{STAT6/cEBP})_2$ was generated by annealing oligonucleotides OLS25 (5'-acttcggacgtcTTCTTATGAACAGGCTGTATTAGCCAACTTCTTATGAACAGGCTGTATTAGCCAACcctgcaggacttcg-3') and OLS26 (5'-cgaagtcctgcaggGTTGGCTAATACAGCCTGTTCATAAGAAGTTGGCTAATACAGCCTGTTCATAAGAAgacgtccgaagt-3'). A second $(O_{STAT6/cEBP})_2$ was PCR-amplified from pWW124 using oligonucleotides OLS27 (5'-acttcgcctgcaggTTCTTATGAACAGGCTGTATTAGCCAACTTCTTATGAACAGGCTGTATTAGCCAACTCGAGCTCGGTACCCGGGTC-3') and OLS19 (5'-acttcggaattcCCGCGG AGGCTGGATCGGTC-3'). The first $(O_{STAT6/cEBP})_2$ was restricted with *Aat*II/*Sbf*I, the second $(O_{STAT6/cEBP})_2$ was restricted with *Sbf*I/*Eco*RI and both fragments were ligated into the corresponding sites (*Aat*II/*EcoR*I) of pWW124. | Schukur et al., 2015[4] |
| pLS16 | $P_{hCMV}$-driven STAT6 expression vector ($P_{hCMV}$- *STAT6*- pA). STAT6 was PCR-amplified from pSTAT6 using oligonucleotides OLS38 (5'-ACTTCGGAATTCGCCACCATGTCTCTGTGGGGTCTG GTC-3', *EcoR*I underlined), and OLS39 (5'-ACTTCGGCGGCCGCTCACCAACTGGGGTTGGCCCTT AG-3', *Not*I underlined), restricted with *Eco*RI/*Not*I and ligated into the corresponding sites (*Eco*RI/*Not*I) of pcDNA3.1(+). | Schukur et al., 2015[4] |
| pDARPin E2_79 | pUC57-derived vector containing N-terminally His-tagged DARPin E2_79 (His-DARPin E2_79). | This work |
| pCH15 | $P_{hCMV}$-driven DARPin E2_79 expression vector ($P_{hCMV}$-$SS_{SEAP}$-*His-DARPin E2_79*-pA). His-DARPin | This work |

| | | |
|---|---|---|
| | E2_79 was PCR-amplified from pDARPin E2_79 using oligonucleotides OCH32 (5'-cgcaagcttacgatgctgctg ctgctgctgctgctgggcctgaggctacagctctccctgggcCATATGAGAGGGTCACATCAC -3'), and OCH27 (5'-cgcgaattctcaCTGCAGGATTTCAGCCAGGTC-3'), restricted with *Hin*dIII/*Eco*RI and cloned into the corresponding sites (*Hin*dIII/*Eco*RI) of pcDNA3.1(+). | |
| pCH23 | $P_{hCMV}$-driven IL13RA1 expression vector ($P_{hCMV}$-*IL13RA1*-pA). IL13RA1 was PCR-amplified from pIL13RA1 using oligonucleotides OCH41 (5'-cgcgaagcttacgATGGAGTGGCCGGCGCGGCTC-3'), and OCH42 (5'-cgcggaattcTCACTGAGAGGCTTTCTTCAG-3'), restricted with *Hin*dIII/*Eco*RI and cloned into the corresponding sites (*Hin*dIII/*Eco*RI) of pcDNA3.1(+). | This work |
| pCH24 | $P_{hCMV}$-driven IL4RA expression vector ($P_{hCMV}$-*IL4RA*-pA). IL4RA was PCR-amplified from pIL4RA using oligonucleotides OCH43 (5'-cgcgggatccacgATGGGGTGGCTTTGC TCTGGG-3') and OCH44 (5'- cgcggaattcCTAAGAGACCCTCATGTATGT-3', *Eco*RI underlined), restricted with *Bam*HI/*Eco*RI and cloned into the corresponding sites (*Bam*HI/*Eco*RI) of pcDNA3.1(+). | This work |
| pCH26 | $P_{STAT6/cEBP}$-driven DARPin E2_79 expression vector ($P_{STAT6/cEBP}$-*$SS_{SEAP}$-His-DARPin E2_79*-pA). $SS_{SEAP}$-His-DARPin E2_79 was PCR-amplified from pCH15 using oligonucleotides OCH47 (5'-cgctctagaacgATGC TGCTGCTGCTGCTGCTG-3', *Xba*I underlined), and OCH48 (5'-cgcaagctttcaCTGCAGGATTTCAGCCAGGTC-3'), restricted with *Xba*I/*Hin*dIII and cloned into the corresponding sites (*Xba*I/*Hin*dIII) of pLS12. | This work |

**Abbreviations**: **DARPin E2_79**, designer ankyrin repeat protein E2_79 codon-optimized for *homo sapiens*; **IL4RA**, human interleukin 4 receptor alpha; **IL13RA1**, human interleukin 13 receptor alpha 1; **His**, His-Tag, 6x polyhistidine tag; **STAT6**, human signal transducer and activator of transcription 6; **$(O_{STAT6/cEBP})_n$**, n tandem repeats of the STAT6- and c/EBP-specific operator; **MCS**, multiple cloning site; **pA**, polyadenylation signal; **PCR**, polymerase chain reaction; **$P_{hCMV}$**, human cytomegalovirus immediate early promoter; **$P_{hCMVmin}$**, minimal version of $P_{hCMV}$; **$P_{lac}$**, promoter of the *E. coli* lactose operon; **$P_{SPA}$**, SCB1-responsive promoter ($O_{papRI}$-$P_{hCMVmin}$); **$P_{STAT6/cEBP}$**, STAT6-responsive promoter ($(O_{STAT6/cEBP})_4$-$P_{hCMVmin}$); **$P_{SV40}$**, simian virus 40 promoter; **$P_{T7lac}$**, phage T7 promoter linked to the *E. coli* lac operator; **SCB1**, *Streptomyces coelicolor* butanolide 1,2-(1'hydroxy-6-methylheptyl)-3-(hydroxymethyl)-butanolide; **SEAP**, human placental secreted alkaline phosphatase; **$SS_{SEAP}$**, SEAP-derived secretion signal.
**Oligonucleotides:** Restriction endonuclease-specific sites are shown in lower case underlined, annealing base pairs are indicated in capital letters and operator sites ($O_{STAT6/cEBP}$) are indicated by underlined capital letters.

## References


1. Rubin, G. M. *et al.* A Drosophila Complementary DNA Resource. *Science* **287**, 2222-2224 (2000).
2. Lennon, G. *et al.* The I.M.A.G.E. Consortium: An Integrated Molecular Analysis of Genomes and Their Expression. *Genomics* **33**, 151-152 (1996).
3. Weber, W. *et al.* Streptomyces-derived quorum-sensing systems engineered for adjustable transgene expression in mammalian cells and mice. *Nucleic Acids Research* **31**, e71-e71 (2003).
4. Shukur, L. *et al.* Implantable synthetic cytokine converter cells with AND-gate logic treat experimental psoriasis. *Science Translational Medicine* **7**, 318ra201 (2015).
